# A comprehensive phenotypic characterization of a whole-body *Wdr45* knock-out mouse

**DOI:** 10.1007/s00335-021-09875-3

**Published:** 2021-05-27

**Authors:** Caroline A. Biagosch, Silvia Vidali, Michael Faerberboeck, Svenja-Viola Hensler, Lore Becker, Oana V. Amarie, Antonio Aguilar-Pimentel, Lillian Garrett, Tanja Klein-Rodewald, Birgit Rathkolb, Enrica Zanuttigh, Julia Calzada-Wack, Patricia da Silva-Buttkus, Jan Rozman, Irina Treise, Helmut Fuchs, Valerie Gailus-Durner, Martin Hrabě de Angelis, Dirk Janik, Wolfgang Wurst, Johannes A. Mayr, Thomas Klopstock, Thomas Meitinger, Holger Prokisch, Arcangela Iuso

**Affiliations:** 1 Institute of Human Genetics, Technische Universität München, 81675 Munich, Germany; 2 Institute of Neurogenomics, Helmholtz Zentrum München, 85764 Neuherberg, Germany; 3 Department of Pediatrics, University Hospital Salzburg, Paracelsus Medical University, 5020 Salzburg, Austria; 4 Institute of Developmental Genetics, Helmholtz Zentrum München, 85764 Neuherberg, Germany; 5 German Mouse Clinic, Institute of Experimental Genetics Helmholtz Zentrum München, 85764 Neuherberg, Germany; 6 Institute of Developmental Genetics, Helmholtz Zentrum München, 85764 Neuherberg, Germany; 7 Institute of Molecular Animal Breeding and Biotechnology, Gene Center, Ludwig-Maximilians-University Munich, 81377 Munich, Germany; 8 German Center for Diabetes Research (DZD), 85764 Neuherberg, Germany; 9 Chair of Experimental Genetics, TUM School of Life Sciences (SoLS), Technische Universität München, 85354 Freising, Germany; 10 Chair of Developmental Genetics, TUM School of Life Sciences (SoLS), Technische Universität München, 85354 Freising, Germany; 11 Deutsches Zentrum Für Neurodegenerative Erkrankungen (DZNE) Site Munich, 81377 Munich, Germany; 12 Munich Cluster for Systems Neurology (SyNergy), Adolf-Butenandt-Institut, Ludwig-Maximilians-Universität München, 81377 Munich, Germany; 13 Department of Neurology, Friedrich-Baur-Institute, Ludwig-Maximilians-University, 80336 Munich, Germany; 14 Present Address: Institute of Molecular Genetics of the Czech Academy of Sciences, Czech Centre for Phenogenomics, Prumyslova 595, Vestec, 252 50 Czechia

## Abstract

Pathogenic variants in the *WDR45* (OMIM: 300,526) gene on chromosome Xp11 are the genetic cause of a rare neurological disorder characterized by increased iron deposition in the basal ganglia. As *WDR45* encodes a beta-propeller scaffold protein with a putative role in autophagy, the disease has been named Beta-Propeller Protein-Associated Neurodegeneration (BPAN). BPAN represents one of the four most common forms of Neurodegeneration with Brain Iron Accumulation (NBIA). In the current study, we generated and characterized a whole-body *Wdr45* knock-out (KO) mouse model. The model, developed using TALENs, presents a 20-bp deletion in exon 2 of *Wdr45*. Homozygous females and hemizygous males are viable, proving that systemic depletion of *Wdr45* does not impair viability and male fertility in mice. The in-depth phenotypic characterization of the mouse model revealed neuropathology signs at four months of age, neurodegeneration progressing with ageing, hearing and visual impairment, specific haematological alterations, but no brain iron accumulation. Biochemically, *Wdr45 KO* mice presented with decreased complex I (CI) activity in the brain, suggesting that mitochondrial dysfunction accompanies Wdr45 deficiency. Overall, the systemic *Wdr45* KO described here complements the two mouse models previously reported in the literature (PMIDs: 26,000,824, 31,204,559) and represents an additional robust model to investigate the pathophysiology of BPAN and to test therapeutic strategies for the disease.

**Supplementary Information:**

The online version contains supplementary material available at 10.1007/s00335-021-09875-3.

## Background

Neurodegeneration with brain iron accumulation (NBIA) comprises a heterogeneous group of disorders sharing the eponymous feature of iron deposition in basal ganglia. The prevalence is 1–3 in 1,000,000, and there are 15 disease-causing genes identified to date, each responsible for a specific subtype of NBIA (Di Meo and Tiranti 2018).

Beta-propeller protein-associated neurodegeneration (BPAN) is a subtype of NBIA and was formerly known as static encephalopathy of childhood with neurodegeneration in adulthood (SENDA). Its old name described this disorder’s main clinical feature of static psychomotor retardation in childhood, followed by a progressive deterioration in adolescence or young adulthood (Hayflick et al. 2013). From the discovery of the gene *WDR45* (Haack et al. 2012; Saitsu et al. 2013), an increasing number of patients were diagnosed. Within the group of NBIA disorders, BPAN shows a unique clinical presentation (Haack et al. 2013). Epilepsy and global developmental delay with impaired speech, motor impairment, and intellectual disability, albeit to a variable extent, typically start early in childhood. Features remain essentially static until early adulthood. Most patients rapidly progress to a severe disability with progressive parkinsonism, dystonia, spasticity, and intellectual deterioration. MRI typically shows hypointense globus pallidus and substantia nigra in T2-weighted MRI (indicating excess iron deposition), a pathognomonic halo sign around the substantia nigra iron in T1-weighted MRI, as well as general cerebral atrophy (Hayflick et al. 2013).

BPAN is an X-linked dominant disorder. The majority of individuals with BPAN are either heterozygous females or hemizygous mosaic males carrying de novo mutations (Haack et al. 2012). However, a few male patients who had inherited pathogenic variant in *WDR45* from the mother are also described (Abidi et al. 2016; Nakashima et al. 2016), suggesting that some germline mutations in *WDR45* might be compatible with life.

*WDR45* encodes the protein WDR45, also known as WIPI4 (WD-repeat domain phosphoinositide-interacting protein 4). It belongs to the WD-repeat protein family, which contains a conserved structural motif of more than 40 amino acids that terminate in tryptophan-aspartic acid (WD) residues. WDR45 consists of seven repeat units, which form a circularized seven-bladed beta-propeller. This secondary structure is responsible for its associated disease name Beta-Propeller Protein-Associated Neurodegeneration. In humans and mice, there are four WIPI proteins, WIPI 1–4. WIPI proteins are involved in cell cycle control, apoptosis, and autophagy (Behrends et al. 2010). They are known to play a central role in detecting the phosphatidylinositol 3-phosphate (PI3P) pool within cells, which is a critical step in the process of autophagy (Proikas-Cezanne et al. 2015). Genetically confirmed BPAN patients’ lymphoblastoid cell lines (Saitsu et al. 2013) and primary fibroblasts (Seibler et al. 2018) consistently show impaired autophagic flux. BPAN lymphoblastoid cell lines showed the accumulation of abnormal early autophagic structures by co-localization of the microtubule-associated protein 1A/1B-light chain 3–phosphatidylethanolamine conjugate (LC3II) with the autophagy-related protein 9 (ATG9A), with the latter, naturally being absent from mature autophagosomes, in contrast to LC3II. Besides altered autophagy, BPAN fibroblasts showed iron overload accompanied by mitochondrial stress and lysosomal dysfunction (Seibler et al. 2018). Moreover, WIPI proteins have been reported to be aberrantly expressed in cancer (Proikas-Cezanne et al. 2004).

A mouse model with a central nervous system (CNS)-specific KO of *Wdr45* revealed neurobehavioral and neurological abnormalities at 11–13 months (Zhao et al. 2015). Neuropathological findings suggested severe neurodegeneration, with eosinophilic spheroids indicating axon swellings. Axon degeneration, swollen mitochondria, and vacuolated structures in different brain regions, as well as lower autophagic activity, were discovered. No iron deposits were spotted. Although the results resembled the human phenotype, the generated mouse had the limitation of presenting with the mutation only in the brain. Recently, two constitutive *Wdr45* KO mouse models were generated (Wan et al. 2020; International Mouse Phenotyping Consortium (IMPC) 2021). The mouse model from Wan and colleagues displayed deficits in spatial learning and conditioning memory and no motor coordination impairment at around six months. During ageing, the whole KO mouse presented few pathological features of BPAN patients, such as iron accumulation and loss of TH^+^ and RBFOX3^+^ neurons. Impaired endoplasmic reticulum (ER) turnover and ER stress were observed in those mice and ascribed to impaired autophagy. The mouse model generated by the IMPC showed only hearing impairment at a preliminary neurological examination, and changes in the body length and bone mineralization. A deep neurological and pathological investigation is lacking.

Here, we report on the generation and extensive characterization of an additional BPAN mouse model in which *Wdr45* has been systemically knocked out using transcription activator-like effector nucleases (TALENs), a new method for a model of BPAN. Our model confirmed many histopathological and neurological findings from the two reported BPAN mouse models and the hearing loss found in the model generated by the IMPC. Furthermore, it provided suggestions about new phenotypic features of germline deficiency in mice and shed light on the involvement of mitochondrial dysfunction in the pathogenesis of BPAN.

## Results

### Viability and fertility of* Wdr45* KO mice

We produced *Wdr45* KO mice by introducing a 20-bp deletion in exon 2 of the gene using the TALENs technique (Fig. S1). This deletion causes a frameshift and is predicted to introduce a premature stop codon 35 amino acids after the initial methionine (Fig. S2a). Two founder mice, a male and a female, were proven to carry the deletion (indicated with #1 and #2 in Fig. S2b). Founder mice were bred with C57Bl/6 N mice resulting in 10 mutants out of 16 animals in the F1 generation (*n* = 6 *Wdr45*^*−/*+^, *n* = 4 *Wdr45*^*−/Y*^). KO confirmation in the F1 generation was achieved by Sanger sequencing (Fig. S2c) and quantitative RT-PCR (Fig. S2D). Commercially available antibodies (168,532, Abcam and AP13313a, Abgent) were unable to detect endogenous Wdr45 protein in various mouse tissues. Therefore, no protein validation of the KO could be provided. Inbreeding of the mutant F1 generation resulted in *Wdr45*^+*/*+^, *Wdr45*^*−/Y*^, *Wdr45*^+*/Y*^, *Wdr45*^*−/*+^*,* and *Wdr45*^*−/ −*^mice in the F2 generation (Fig. S1). Having obtained viable mice with the five possible genotypes proved that systemic depletion of a wild-type *Wdr45* allele impairs neither viability nor male fertility in *Wdr45* KO mice.

### Progression of neurodegeneration in *Wdr45* KO mice

**Fig. 1:**
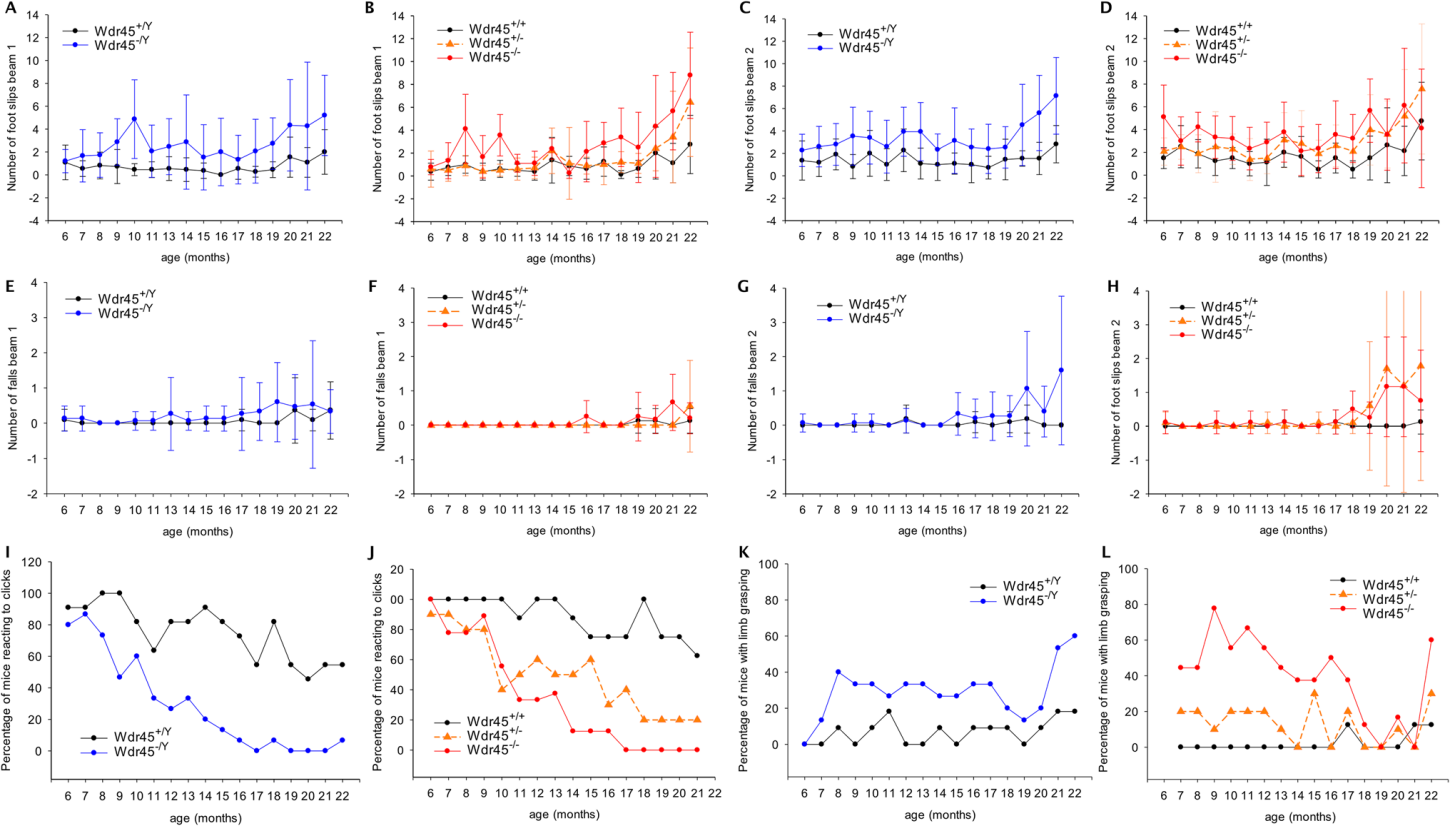
Neurologic deterioration in *Wdr45* KO mice. **a**–**d** Males *Wdr45*^*−/Y*^ (A, C), as well as females *Wdr45*^*−/−*^ (**b**, **d**) of the progression cohort, made more slips compared to controls on both beams tested (linear mixed-effects model; genotype effect *p* < 0.001 each). **e**–**h** The number of falls from the different beams was not altered on beam 1 (**e**, **f**) but mildly increased for beam 2 (**g**, **h**; linear mixed-effects model; genotype effects *Wdr45*^*−/Y*^ *p* < 0.05; *Wdr45*^*−/*+^ *p* < 0.05; *Wdr45*^*−/−*^ n.s.). **i**, **j**) Decreasing numbers of both sexes’ mutant mice reacted to a clickbox (linear model; genotype *p* < 0.001 each). **k**, **l**) More mutants showed limb grasping compared to controls (linear model; genotype effects *p* < 0.001 for *Wdr45*^*−/Y*^ and *Wdr45*^*−/−*^, *p* < 0.05 for *Wdr45*^*−/*+^). The number of mice used for the tests was as follows: *Wdr45*^*−/*+^ *n* = 10, *Wdr45*^*−/−*^ *n* = 9, *Wdr45*^+*/*+^ *n* = 8, *Wdr45*^*−/Y*^ *n* = 15, *Wdr45*^+*/Y*^ *n* = 11

Mice from the progression cohort—setup to evaluate the disease progression in mutant mice over three years’ time—were regularly checked for neurological impairment for 22 months, with the first check starting at five months of age. Higher body weight was observed in mutant animals of both sexes (at 22 months: 40 g for *Wdr45*^+*/Y*^ vs 44 g for *Wdr45*^*−/Y*^; 36 g for *Wdr45*^+*/*+^ vs 39 g for *Wdr45*^*−/*+^ and 43 g for *Wdr45*^*−/−*^). Balance beam analysis pointed out that mutants (*Wdr45*^*−/Y*^, *Wdr45*^*−/*+^, *Wdr45*^*−/−*^) of both sexes made more slips on the beams (Fig. 1a–d, lme: genotype effect each *p* < 0.001 but *Wdr45*^*−/*+^ n.s. at first beam; age effect *p* < 0.001). The number of falls also increased with age (lme; age effect *p* < 0.001; Fig. 1e–h). *Wdr45*^*−/Y*^ and *Wdr45*^*−/*+^ mice experienced slightly more falls from the second beam than age-matched and sex-matched wild-type animals (*p* < 0.05; Fig. 1g, h). A modified SHIRPA analysis revealed that both male and female mutants were more prone to show tremors with significant differences at 20 months of age (number of animals with tremor: 10/10 *Wdr45*^*−/−*^, 8/10 *Wdr45*^*−/*+^*,* and 10/10 *Wdr45*^*−/Y*^ vs 4/10 *Wdr45*^+*/*+^ and 6/10 *Wdr45*^+*/Y*^; Fisher’s Exact test). A clickbox test revealed a progressive hearing loss in KO mutants of both sexes starting already at a young age. By the age of 17 months, fewer mice of both *Wdr45*^*−/Y*^ and *Wdr45*^*−/−*^ reacted to the clickbox (linear model; genotype effect *p* < 0.001), while for *Wdr45*^*−/*+^*,* some animals reacted till 22 months of age (linear model; *p* < 0.001; Fig. 1i, j). Limb grasping occurred in mutants more often than in wild-type animals (linear model: genotype effects *Wdr45*^*−/Y*^ *p* < 0.001; *Wdr45*^*−/*+^ *p* < 0.05; *Wdr45*^*−/−*^ *p* < 0.001; Fig. 1k, l). Body position, passive behaviour, transfer arousal, gait, tail elevation, trunk curl, touch escape, urination, and grip strength showed no abnormalities (not shown).

**Fig. 2:**
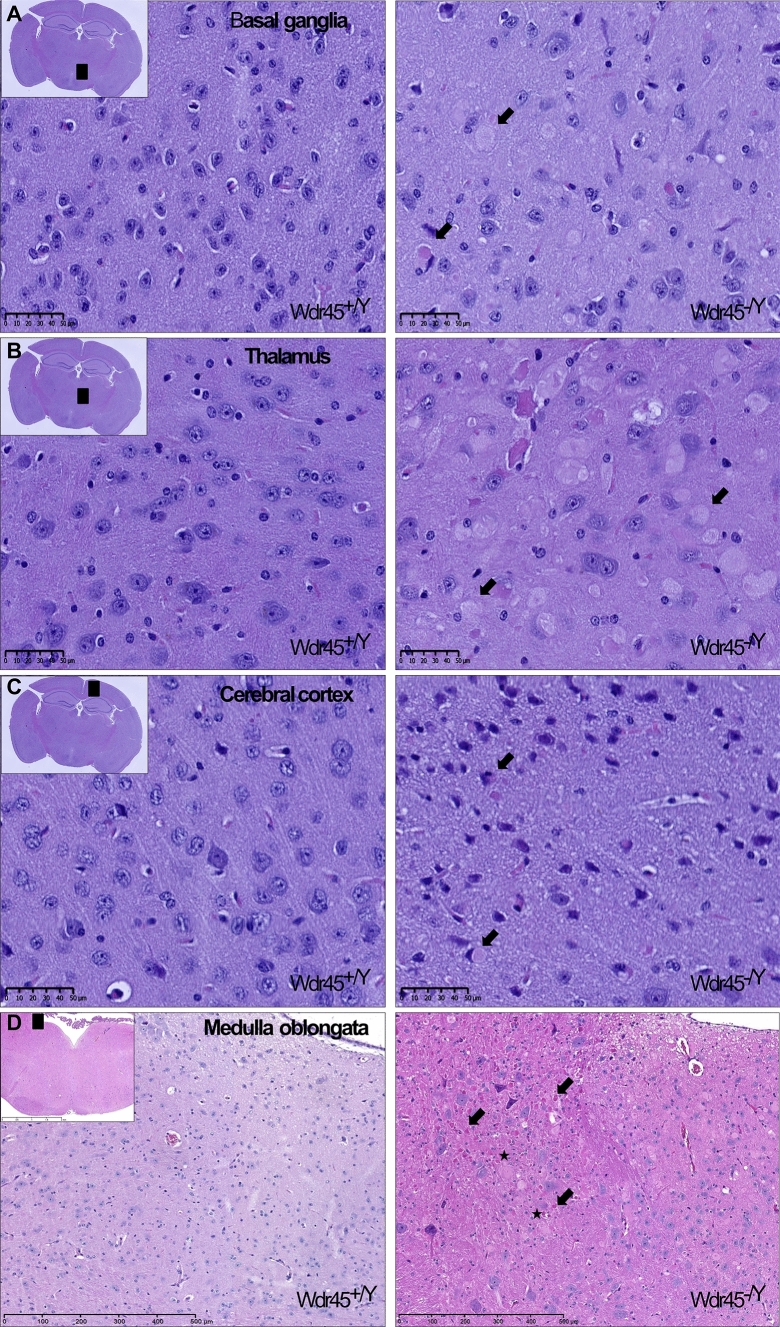
H&E staining in representative tissue sections from 18-month-old *Wdr45*^+*/Y*^ and *Wdr45*^*−/Y*^ mice. **a**–**d** Black squares in the insets indicate the relevant brain area zoomed in the figure. Arrows indicate eosinophil spheroids, while asterisks indicate swollen structures potentially showing degenerated neurons in basal ganglia, thalamus, cerebral cortex, and medulla oblongata of *Wdr45* KO mice. Scale bar represents 50 µm for figures A-C; 500 µm for D

**Fig. 3:**
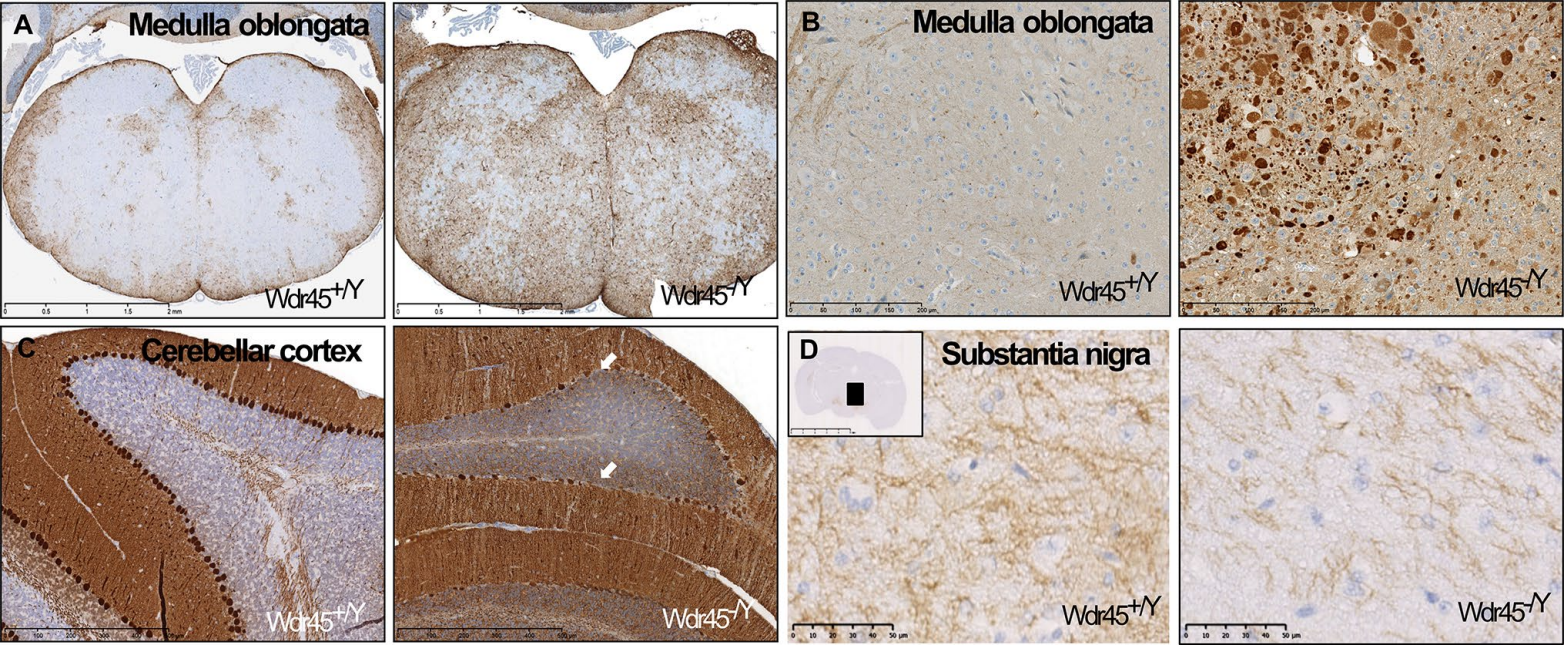
Immunostaining in representative tissue sections from 18-month-old *Wdr45* ^+*/Y*^ and *Wdr45*^*−/Y*^ mice. **a** GFAP staining and **b** Ubiquitin staining in medulla oblongata; **c** Calbindin staining of the cerebellar cortex; **d** Dopamine staining of substantia nigra. Scale bar represents 50 µm for figures A- B and 500 µm for figure D

Neuropathological examination in 53 mice from 4 to 18 months of age (mutants: *n* = 16 *Wdr45*^*−/Y*^, *n* = 10 *Wdr45*^*−/−*^, *n* = 19 *Wdr45*^*−/*+^; Wild-types: *n* = 7 *Wdr45*^+*/*+^; *n* = 1 *Wdr45*^+*/Y*^ mice) revealed pathological signs in all *n* = 45 mutant mice already at 4 months of age. Eosinophil spheroids and swollen structures, mostly due to degenerating neurons, were found in the basal ganglia (Fig. 2a), thalamus (Fig. 2b), cerebral cortex (Fig. 2c), medulla oblongata (Fig. 2d), ascending and descending fibres of the spinal cord, and deep cerebellar nuclei of mutant animals (not shown). Neuropathological signs were consistently present in older animals (Fig. S3). Of note, none of the wild-type animals presented brain abnormalities even at 18 months of age. In order to investigate potentially accumulating substances within swollen or degenerating structures, histochemical and immunohistochemical staining of medulla oblongata were performed. GFAP staining of medulla oblongata showed marked astrocytosis and astrogliosis (Fig. 3a). Iron staining, both Fe^2+^ and Fe^3+^, did not reveal any significant iron accumulation in *Wdr45* KO mice. The iron level was also checked in erythropoiesis organs, but it was also not altered in these tissues (data not shown). No lipofuscin accumulation was shown by the Kluver–Barrera staining nor polysaccharides such as glycogen, glycoprotein, or glycolipids by the PAS staining. LC3II staining and SDHA staining revealed normal cytoplasmic localization in neurons of the medulla oblongata (data not shown), although numerous ubiquitin-positive aggregates were present in the mutant medulla oblongata (Fig. 3b). Calbindin staining revealed an impaired Purkinje cell layer of the mutant cerebellar cortex (Fig. 3c). Dopamine staining revealed a reduced number of neuroaxonal fibres in the substantia nigra of *Wdr45* KO animals (Fig. 3d).

### In-depth phenotypic characterization of *Wdr45* KO mice

**Fig. 4:**
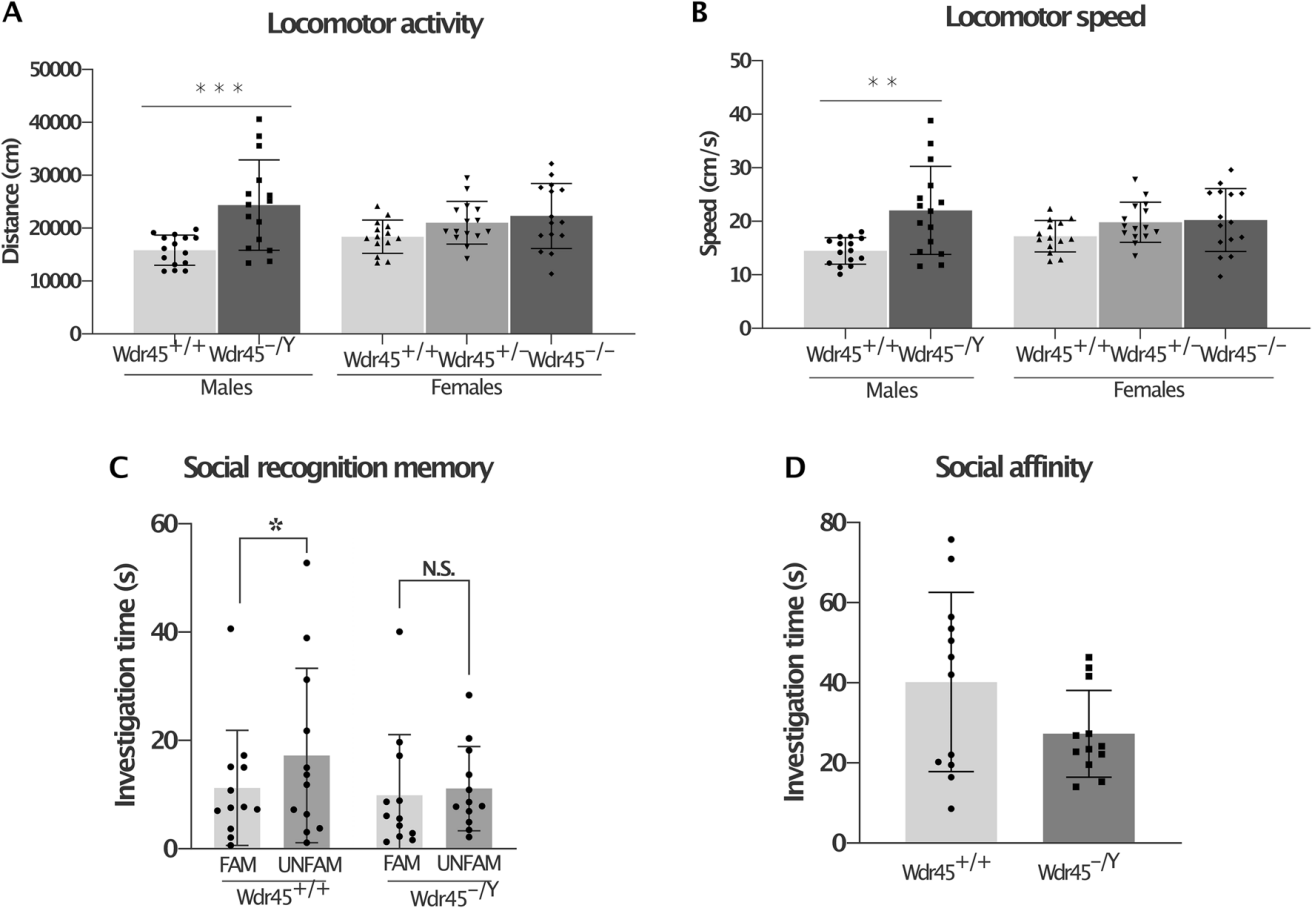
Locomotor activity and social discrimination in *Wdr45* KO mice. There was increased total distance travelled **a** and locomotor speed **b** by the male *Wdr45*^*−/Y*^ mice in the open field at 11 months of age. A similar pattern was visible in especially the female *Wdr45*^*−/−*^ mice. **p < 0.01, ***p < 0.001, 1-way ANOVA with post hoc *Tukey’s* test. Social discrimination memory was impaired in the male *Wdr45*^*−/Y*^ mice at 12.5 months of age (**c**). During the test phase, the *Wdr45*^*−/Y*^ mice spent equivalent times investigating familiar (“FAM”) vs unfamiliar (“UNFAM”) stimulus animals, while the control mice spent significantly more time investigating UNFAM mice. A pattern of decreased social affinity was also evident (**d**). Bars indicate standard deviation; *paired *t* test: *p* < 0.05. The number of mice used for the tests was as follows: open field: male *Wdr45*^+*/*+^ *n* = 15, *Wdr45*^*−/Y*^ *n* = 15, female *Wdr45*^+*/*+^ *n* = 14, *Wdr45*^±^ *n* = 15, *Wdr45*^*−/−*^ *n* = 15; social discrimination: male *Wdr45*^+*/*+^ *n* = 12, *Wdr45*^*−/Y*^ *n* = 12

The phenotyping cohort consisting of 127 animals (*Wdr45*^*−/*+^ *n* = 25, *Wdr45*^*−/−*^ v17, *Wdr45*^+*/*+^ *n* = 21, *Wdr45*^*−/Y*^ *n* = 26, *Wdr45*^+*/Y*^ *n* = 38) was used to evaluate neurological functions as well as to investigate non-neurological traits in *Wdr45* KO mice. All investigations were conducted on mice between 11 and 16 months of age. Mice from the age of 11 months underwent a series of behavioural assays to assess the following behaviours: locomotor activity and speed in the open field (spontaneous reactions to novelty), social discrimination memory (Test phase data: time spent with familiar compared to time spent with the unfamiliar animal), and social affinity (Sample phase data: time spent with the familiar animal). *Wdr45*^*−/Y*^ males travelled a greater distance with greater speed compared to age-matched and sex-matched controls (1-way ANOVA distance effect: *F*(4,69) = 5.68, *p* = 0.0005, post hoc *Tukey’s* test, male *Wdr45*^+*/Y*^ vs. *Wdr45*^*−/Y*^ *p* < 0.0001, speed effect: *F*(4,69) = 4.93, *p* = 0.002, post hoc *Tukey’s* test, male *Wdr45*^+*/*+^ vs. *Wdr45*^*−/Y*^ *p* < 0.01 Fig. 4a, b). A similar hyperactivity pattern was evident in both the female *Wdr45*^*−/*+^ and *Wdr45*^*−/−*^ mice compared to the age-matched and sex-matched controls. In the social recognition test for evaluation of social memory, *Wdr45*^*−/Y*^ males (only males tested) spent the same amount of time with familiar and unfamiliar animals (no significant difference in *Wdr45*^*−/Y*^ using paired one-tailed *t* test: *Wdr45*^+*/*+^: *t*(12) = 2.07, *p* = 0.03, *Wdr45*^*−/Y*^: *t*(12) = 0.36, *p* = 0.36) thus revealing impaired social recognition memory compared to controls (Fig. 4c, d).

Tremors were observed in 12-month-old mice in 9/15 *Wdr45*^*−/−*^, 1/15 *Wdr45*^*−/*+^, and 9/15 *Wdr45*^*−/Y*^ mutant versus 1/15 *Wdr45*^+*/*+^ and 2/15 *Wdr45*^+*/Y*^ wild-type animals at 12 months of age.

ABR test in 12-month-old mice revealed reduced hearing sensitivity in *Wdr45*^*−/*+^ females while *Wdr45*^*−/−*^ females and *Wdr45*^*−/Y*^ males were virtually deaf (Fig. S4A). Grip strength at that age was not different between genotypes (Fig. S4B).

The balance beam analysis in 12-month-old mice showed that both *Wdr45*^*−/−*^ females as well as *Wdr45*^*−/Y*^ males made significantly more foot slips on all beams compared to controls and required more time to traverse the beams (Fig. S4c, d; Wilcoxon rank-sum test; genotype effects *p* < 0.01 each). There was no global effect on stops or falls (data not shown). The beam ladder analysis in 15-month-old mice showed that *Wdr45*^*−/y*^*,* as well as *Wdr45*^*−/*+^ mutants, needed more time to traverse the horizontal ladder compared to wild-type mice (Wilcoxon rank-sum test; *p* < 0.001) while *Wdr45*^*−/*+^ females performed similarly to controls (Fig. S4e). Forepaw slips were similar between genotypes, but hindpaw slips occurred more often in *Wdr45*^*−/Y*^ and *Wdr45*^*−/−*^ mice (Fig. S4f, g; Wilcoxon rank-sum test; *p* < 0.01). Again, *Wdr45*^*−/*+^ females performed similarly to controls.

**Fig. 5:**
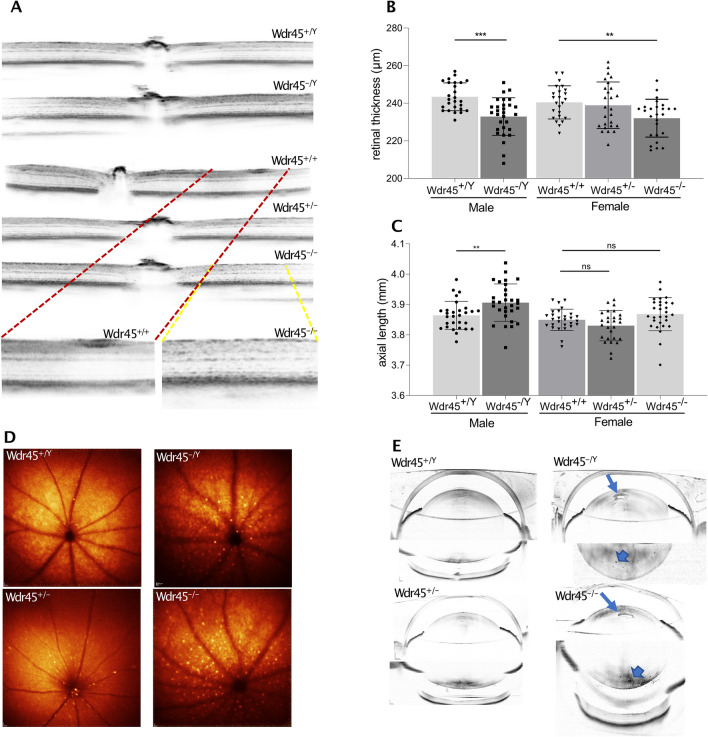
Ophthalmic examination in *Wdr45* KO mice. **a** SD-OCT imaging of the retinal layers indicating granular-like alterations in the retinal layers of the female *Wdr45*^*−/−*^ and male *Wdr45*^*−/Y*^ mice from the progression cohort. Magnification of the granular-like alterations in the insets. **b** SD-OCT evaluation of the total retinal thickness. **c** Biometric measurement of the eye-axial lengths. The bar charts show pooled data of both eyes for each animal. The genotype effect was evaluated with the Wilcoxon rank-sum test (homozygous females/hemizygous males vs wild-type). **d** BAF funduscopy of the inner retinal layers in female Wdr45^−/−^ and male *Wdr45*^*−/y*^ mice from the F8–F9 generations. Accumulation of hyperfluorescent profiles is evident. **e** SD-OCT images of the eye lens in female *Wdr45*^*−/−*^ and male *Wdr45*^*−/y*^ mice from the F8–F9 generations showing an accumulation of protein aggregates in the lens anterior cortex (arrow). Additional alterations are seen as darker spots in the posterior lens cortex (arrowheads). The number of mice used for the tests was as follows: *Wdr45*^+*/*+^ *n* = 12, *Wdr45*^*−/−*^ *n* = 13 *Wdr45* ^+*/Y*^ *n* = 13, and *Wdr45 *^*−/Y*^ *n* = 15 from the F4 cohort; *Wdr45*^+*/*+^ *n* = 6, *Wdr45*^*−/−*^ *n* = 2, *Wdr45* + ^*−/Y*^ *n* = 3, and *Wdr45 *^*−/Y*^ *n* = 5 from the F8–F9 cohort

Ophthalmic examination in mice from the phenotyping cohort (F4 generation) at around 13 months of age showed severe retinal degeneration in 5/13 *Wdr45*^*−/−*^ and 5/15 *Wdr45 *^*−/Y*^ mice, with clear altered OCT signal depicted in the SD-OCT images as a darker dotted pattern (Fig. 5a). At the retinal organization level, a reduction in the total retinal thickness was measured (Fig. 5b). The analysis of the biometric parameters identified an increment of the axial eye length, a reduced thickness of the cornea, and a deeper anterior chamber in mice of both sexes (Fig. 5c; Table S1). Ophthalmic examination in 2 *Wdr45*^*−/−*^ and 5 *Wdr45*^*−/Y*^ mice from later generations (F8–F9) between 10 and 14 months of age revealed a reduction in the total retinal thickness in both *Wdr45*^*−/−*^ females. None of the 5 *Wdr45*^*−/Y*^ males presented such reduction. However, mice from both sexes presented with the same dotted pattern in the retinal layers observed in mice from the F4 generation (Fig. S5a, b). Also, lipofuscin, the pathogenic marker for retinal degeneration (Ueda et al. 2016; Nafar et al. 2020), accumulated in the retina of all mice from the F8–9 generations. (Fig. 5d). Imaged lenses of the F8–F9 *Wdr45*^*−/−*^ and *Wdr45*^*−/Y*^ mice showed alterations like protein aggregates accumulation into the lens cortex matrix (Fig. 5e).

**Fig. 6:**
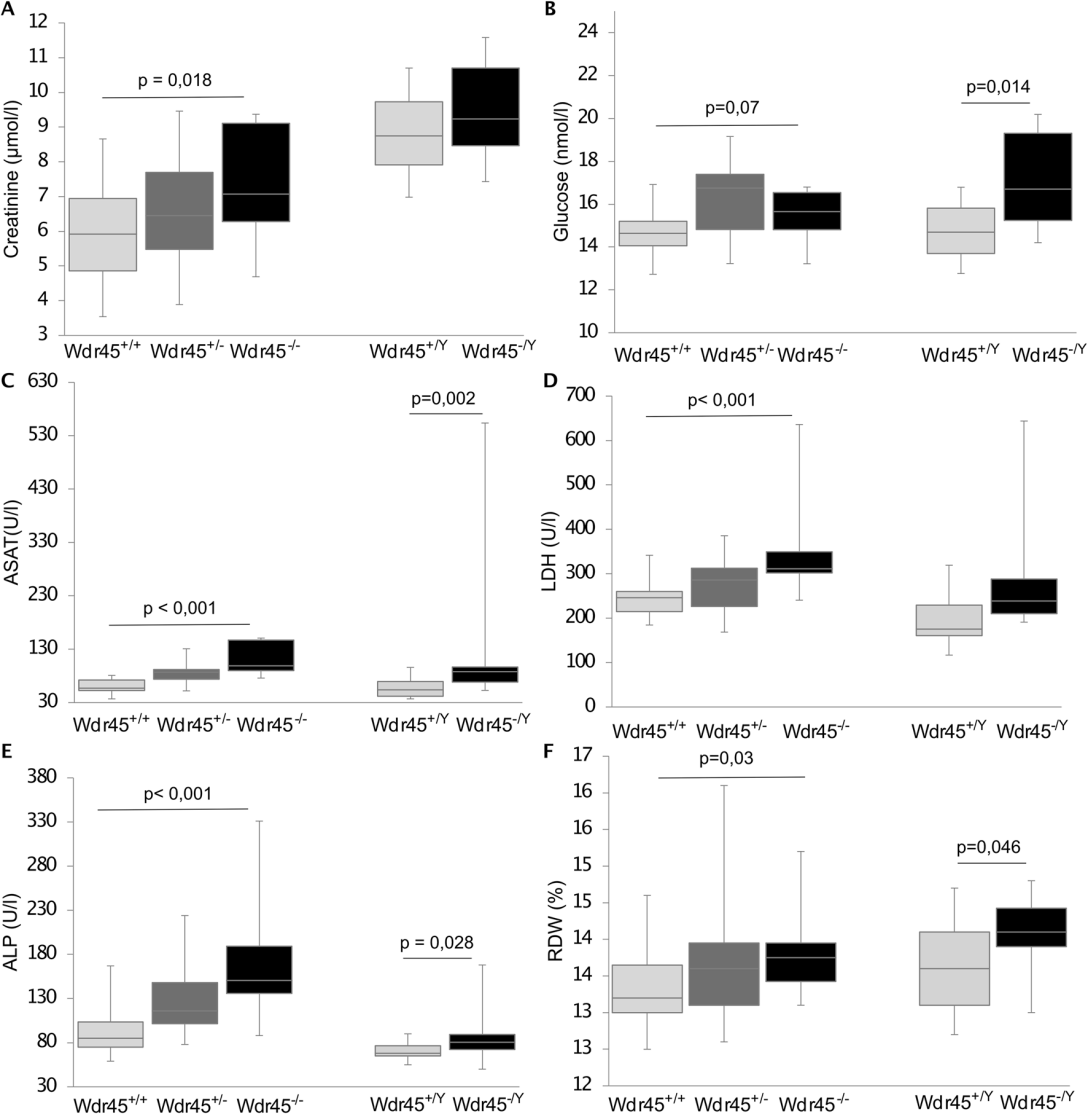
Clinical chemistry and haematology investigation. **a** Creatinine, **b** glucose, **c** aspartate aminotransferase (ASAT), **d** lactate dehydrogenase (LDH), **e** alkaline phosphatase (ALP), and **f** red blood cells distributed widths (RDW) were measured in 14-month-old mice. Tests for genotype effects were made using the Wilcoxon rank-sum test (homozygous females/hemizygous males vs wild-type). The number of mice used for the tests was as follows: *Wdr45*^*−/*+^ *n* = 15, *Wdr45*^*−/−*^ *n* = 14, *Wdr45*^+*/*+^ *n* = 15, *Wdr45*^*−/Y*^ *n* = 14, *Wdr45*^+*/Y*^ *n* = 15

Clinical chemistry in 14-month-old mice revealed increased plasma creatinine and glucose levels as well as aspartate aminotransferase (ASAT), lactate dehydrogenase (LDH), and alkaline phosphatase (ALP) activities in female *Wdr45*^*−/−*^ and in male *Wdr45*^*−/Y*^ mutants compared to age-matched and sex-matched controls (Fig. 6a–e; Table S2), while plasma lactate concentrations were decreased in these animals compared to wild-type controls (Table S2). The trend was the same in female *Wdr45*^*−/*+^ mice, although the difference was not statistically significant for each of these parameters. Furthermore, we observed decreased plasma proteins (TP, albumin, TIBC) predominantly in male mutants and decreased alpha-amylase activity and triglycerides levels in male mutants only (Fig. S6a–e; Table S2). Additionally, slightly increased plasma iron concentration and increased transferrin saturation were observed in *Wdr45* KO animals (Fig. S6f, g; Table S2). In the glucose tolerance test, a mild delay of glucose clearance was observed for *Wdr45*^*−/−*^ females, and the same trend was also seen for *Wdr45*^*−/*+^ females. No significant differences between genotypes were detected in males (not shown).

Haematological data showed elevated values for red blood cells distributed widths (RDW) in *Wdr45* KO animals, indicating slightly increased anisocytosis (Fig. 6f; Table S2). Increased leukocyte (WBC) and erythrocyte counts (RBC), with accordingly elevated haemoglobin (HGB) levels and hematocrit (HCT) values, were observed only in *Wdr45*^*−/Y*^ mutants, possibly indicating hemoconcentration due to altered water balance in these animals (Figs. S6h, k; Table S2).

No dysmorphic features were present in *Wdr45* KO mice; bones and cartilages were normal. There were no apparent changes in the metabolism measured by indirect calorimetry. The body surface temperature was in the normal range, as well as the body mass, body fat, and lean mass. Cardiovascular parameters were also not in the pathological range. There were no indications of immunological reactions or allergies (data not shown). Mutant and wild-type males showed comparable body weight (at 16 months, 41 g for *Wdr45*^+*/Y*^ vs 42 g for *Wdr45*^*−/Y*^) while female *Wdr45*^*−/−*^ showed increased body weight compared to *Wdr45*^+*/*+^ females (at 16 months, 30 g for *Wdr45*^+*/*+^ vs 38 g for *Wdr45*^*−/−*^).

### Mitochondrial function is affected in the brain of *Wdr45* KO mice

**Fig. 7:**
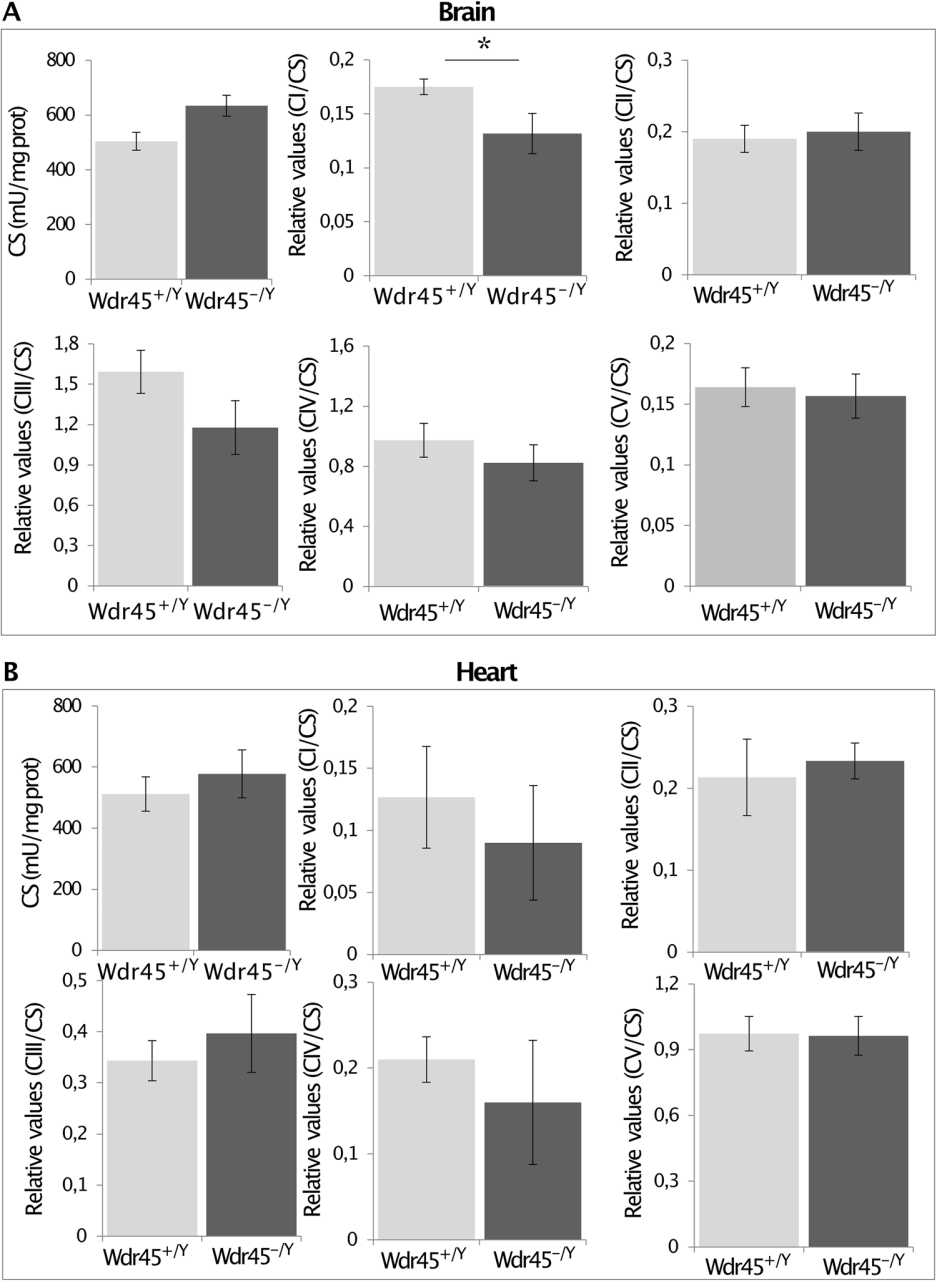
The respiratory chain complexes I–V activity and the citrate synthase (CS) measured in **a** brain and **b** heart of mice from the progression cohort. Data on the activity of the complexes I–V are normalized to the CS. Data are represented as mean $\pm$ SEM. The number of mice used for the brain’s respiratory chain activities measurements was *n* = 6, while for the measures in the heart, mice were n = 3. Mann–Whitney test, *p < 0.05

We performed activity measurement of the citrate synthase (CS) and the five OXPHOS complexes in male mutants between 12 and 18 months of age. Measurements were performed on whole brain extract and the heart muscle. The CS activity showed a trend towards an increase in mutant mice’ brain compared to age-matched controls (Fig. 7a; Mann–Whitney test, *p* = 0.052). When the activities of the respiratory chain complexes I, II, III, IV, and V were normalized to the CS activity in the brain, the ratio complex I/CS in *Wdr45* KO mice was significantly lower compared to that in control mice (Fig. 7a; Mann–Whitney test, *p* = 0.0235). The ratio of the other complexes of the respiratory chain (II to V) remained unaffected (Fig. 7a). Differently from the brain, the CS activity in the heart of *Wdr45* KO mice was comparable to that of wild-type animals (Fig. 7b; Mann–Whitney test, *p* = 0.700), and the activity of the respiratory chain complexes I to V normalized to that of the CS was also comparable between wild-type and *Wdr45* KO mice (Fig. 7b).

## Discussion

BPAN is an X-linked dominant disorder associated with pathogenic variants in the gene *WDR45*. Most of the cases do not inherit the variants but gain them de novo during embryogenesis. Affected individuals are prevalently females, suggesting that *WDR45* mutations negatively affect embryo development in males. De novo mutations in males and females and skewed X-chromosome inactivation in females result in a mosaic distribution of *WDR45* mutations in tissue and organs, thus giving rise to an ample spectrum of clinical features in BPAN patients ranging from highly disabling forms to asymptomatic ones.

In our study, we generated constitutive germline *Wdr45* KO mice. The offspring of the *Wdr45* KO mice had a sex ratio equal to 1, suggesting the absence of male-specific lethality. Moreover, homozygous *Wdr45* KO females and hemizygous *Wdr45* KO males exhibited overlapping phenotypes and comparable readouts in all tests. Only heterozygous *Wdr45* KO females showed a milder presentation compared to homozygous females and hemizygous males, behaving as wild-type animals in some tests, thus reflecting the fact that the mutated X chromosome of this mutant group was randomly inactivated in some cells/tissues. Furthermore, regardless of the genotype, all mutants were fit for breeding, differently from what happens in humans, where most BPAN female patients are considered incapable of reproducing due to the severity of the phenotype. The absence of male lethality and the overall fitness to breed of constitutive *Wdr45* KO animals suggests that mice compensate for loss-of-function mutations in *Wdr45* better than humans.

Looking more specifically into neurodegeneration, *Wdr45*^*−/−*^ and *Wdr45*^*−/Y*^ mice showed significant alterations in motor skills—as revealed by the beam and ladder tests—consistently observed both in the progression and in the phenotyping cohorts. The motor skills’ impairment was initiated at five months of age and progressed during ageing, as indicated by tremors and limb grasping in the older mice. Likewise, delay in a motor milestone in early childhood, often progressing in parkinsonism in early adulthood, is a key presentation of BPAN patients. The attestation of normal grip strength in *Wdr45* KO animals excluded any damage in the skeletal muscle tissue and strengthened the conclusion that motor skills alterations were due to neurological impairment. Epilepsy, a feature most common in the first decade of life in BPAN patients, and dystonia, a feature typical of early adulthood, were instead not displayed by *Wdr45* KO mice at any age. Hearing loss, likely confounded by the patients’ marked cognitive decline and therefore reported only in two BPAN cases (Rathore et al. 2014; Kaleka et al. 2019), was consistently observed in *Wdr45* KO mice both from the progression cohort and phenotyping cohort, as revealed by the clickbox and ABR tests.

Features of autism spectrum disorder often present in BPAN children were also documented in *Wdr45* KO mice. In particular, male animals exhibited autism-like characteristics such as decreased social memory, and a pattern of decreased social affinity and disordered sensory processing manifested as hyperactivity in response to a novel mildly stressful environment. Although the investigation was performed in 12-month-old mice, we cannot exclude that autism-like behaviour was already present at a younger age.

The neuropathology was consistent with the neurobehavioral presentation. Several brain regions with functional specialization were shown to be affected in our *Wdr45* KO mice. For example, basal ganglia, thalamus, and cerebellum are associated with motor coordination; the caudate nucleus, cortex, and cerebellum are involved in learning and memory, and the spinal cord in transmitting sensory signals from peripheral regions to the brain. Affected cell types ranged from glial cells, astrocytes to neurons, and Purkinje cells, with the most characteristic finding being axonal swellings. Pathological signs were consistently observed in KO mice between 4 and 18 months of age, with four months being the first investigation age. Although we did not explore the possibility that pathological signs were already present in younger mice, the presence of spheroids and degenerated neurons at four months of age could serve as an early clinical endpoint in future therapeutic approaches in mice. No iron deposition was detected in the brain of *Wdr45* KO animals, even in the oldest animals, using standard histochemical methods. Even though one BPAN case with a demonstrable mutation in *WDR45* and no iron accumulation (Hoffjan et al. 2016) and another case with the appearance of iron only later in the course of the disease (Okamoto et al. 2014) have been reported, iron accumulation is usually detected as a radiological sign at the same time or soon after the clinical symptoms of progressive dystonia-parkinsonism appear. Therefore, additional investigation with more sensitive methods such as inductively coupled plasma mass spectrometry (ICP-MS) might be useful to ascertain the absence of iron in the substantia nigra of the *Wdr45* KO mice.

As a systemic KO, we questioned whether other organs were affected besides the brain. We observed a few alterations in clinically relevant organ systems. The eye morphology and vision were affected in the *Wdr45 *^*−/−*^ and *Wdr45 *^*−/Y*^ mice of the phenotyping cohort. Despite all retina layers were in place, they presented with granular alterations likely due to the accumulation of proteinaceous debris. As the eye investigations were performed in animals from an early generation (F4), to exclude any confounding effect from the residual genetic FVB background—that is a *retinal degeneration 1* (*rd1*, *rodless*, Pde6b^rd1^) mouse (Grubb et al. 2009)—the ophthalmological investigation was repeated in a small group of animals from later cohorts (F8–F9). Alterations were consistently observed in the older *Wdr45* KO mice as well, suggesting that ocular vision in BPAN patients might be more common than reported (Tiedemann et al. 2018). The metabolic blood parameters hint towards cellular energy metabolism effects, especially of muscle cells, liver or renal function, and cellular integrity of muscle, liver, or blood cells. Glucose tolerance test results suggested mild effects on the regulation of systemic glucose metabolism. Based on the chemical chemistry data suggesting a likely involvement of the energy metabolism of the muscle cells and based on a recent paper reporting on mitochondrial stress as a consequence of *WDR45* deficiency (Seibler et al. 2018), we performed biochemical investigations of the mitochondrial function in the muscle (heart) and brain tissues of the *Wdr45* KO mice. The analysis revealed a brain-specific impairment of the respiratory chain complex I of the *Wdr45* KO mice when the activity of complex I was normalized to the activity of the citrate synthase. The citrate synthase activity tendentially higher in the brain of *Wdr45* KO mice compared to wild-type animals seems to correlate with findings in yeast, where the deletion of autophagy genes was shown to induce mitochondrial dysfunction and accumulation of damaged mitochondria (Zhang et al. 2007; Diogo et al. 2018), as well as findings in mammalian fibroblasts, where autophagy inhibition was shown to lead to an increase of mitochondrial mass (Wang et al. 2008; Wan et al. 2020) and findings in the *Wdr45*-deficient mouse, where mitochondria accumulated in swollen axons (Wan et al. 2020). The brain specificity of the mitochondrial defect might be caused by a different impact of *WDR45* deficiency on dividing and non-dividing cells. While dividing cells can dilute dysfunctional mitochondria, neuronal cells accumulate them, leading to neurodegeneration (Ravikumar et al. 2002; Webb et al. 2003; Berger et al. 2006). Future studies are planned to validate the proposed molecular mechanisms linking *Wdr45* deficiency to complex I defect in the *Wdr45* KO mice.

To date, four different BPAN mouse models have been generated: a brain-specific *Wdr45* KO mouse by Zhao et al. (Zhao et al. 2015; Ji et al. 2020) and three whole-body KOs by Wan et al. (2020), by the IMPC (International Mouse Phenotyping Consortium), and by Biagosch et al. (current study). The four models have been developed using different genetic engineering strategies (Cre-Lox by Zhao (Zhao et al. 2015), CRISPR-Cas by Wan and by the IMPC, TALENs by Biagosch). Although a strict comparison among these models cannot be performed, as animals were characterized by batteries of tests different in each study, all models presented with neurobehavioral phenotypes, albeit with different ages of onset. We have summarized the disease phenotypes of the various models in Table S3.

Motor coordination problems were recorded early in life in our mouse model (5 months of age) and in the systemic KO by Wan et al. (6 months of age), while the brain-specific KO displayed motor problems around 11–13 months of age; memory problems were recorded around 12 months of age in our model and the brain-specific KO, and earlier in life (6 months of age) in the whole-body KO by Wan et al. None of the models showed spontaneous seizures. However, the whole-body KO by Wan et al. showed pilocarpine-induced seizures with increased severity when compared to wild-type mice. Visual acuity was impaired in our model (10–13 months of age) while no information is available from the other models. Auditory function was impaired in our model (5–22 months of age) similarly to the model generated by the IMPC, while no information is available from the other models.

Pathology signs were detected at four months of age in our model and much later in the other two models (13 months in the brain-specific KO and 16 months in the systemic KO by Wan et al.). Iron was not detected in our model and the brain-specific KO but was reported to accumulate in 16-month-old KO mice by Wan et al. No information is available on motor coordination, locomotor activity, iron, and pathology signs in the systemic KO mouse from the IMPC. Memory and social discrimination seem to be normal in this mouse model.

In our study, animals of both sexes were investigated, keeping homozygous females separated from heterozygous females in all tests to point out possible X-chromosome dosage compensation. In Zhao’s study, only male animals were used to exclude the oestrous cycle’s potential effects on mice performances. In Wan’s study, an equal number of males and females was used, without specifying the females’ genotype in the tests. In the IMPC measurements, mostly male were tested.

Systemic manifestations of *Wdr45* deficiency were not investigated in Wan’s model. Body weight, length, composition, bone mineral content, and cardiovascular parameters were instead investigated in the mouse generated by the IMPC and decreased body length and bone mineral content in homozygous females and reduced bone area in homozygous males were shown. We extended the systematical investigation of our mouse model to all organs. The analysis pointed out a few clinical parameters such as chemical chemistry (glucose, ASAT, LDH, ALP) and haematology values (RDW) that could potentially be used as biomarkers of BPAN in the perspective of using the BPAN model for treatment approaches, as they are easy to access in living animals. For instance, increased levels of ASAT and LDH have been recently reported in a group of BPAN patients and suggested by Belohlavkova and colleagues as possible biomarkers of the disease (Belohlavkova et al. 2020).

Thus, the systemic *Wdr45* KO described here complements the three models already available and represents a robust model to test therapeutic approaches and investigate the pathophysiology of BPAN, already providing preliminary data on the correlation between *Wdr45* deficiency and the mitochondrial function in the brain.

## Methods

### Generation of *Wdr45* KO mice

*Wdr45* KO mice were generated using the Transcription Activator-Like Effector Nucleases (TALEN) approach according to the established protocols (Boch et al. 2009; Zhang et al. 2011). The system was designed to introduce mutations in exon 2 of *Wdr45* (NM_001290792.1, GRCm38.p6). Oligonucleotides were codon-optimized for murine usage (GenScript, New Jersey, USA). Before microinjection, TALEN efficiency was tested in vitro using reporter assays (Gal reporter gene assay, Roche Applied Science, Germany; High-sensitivity luciferase reporter gene assay, Roche Applied Science, Germany). *Wdr45* KO mice were generated as shown in Fig. S1. Females of the inbred strain FVB were mated with C57Bl/6 N males. The resulting fertilized oocytes were collected, and TALENs were injected into the larger male pronucleus. Injected embryos were implanted into pseudopregnant CD-1 foster mothers, giving birth to animals with FVB and C57Bl/6 N mixed background. Sanger sequencing using the following primers Forward: CTTCAGAGAGGACACTGGGG and Reverse: TCAGGGTATACGTGGGAAGG identified a 20-bp deletion in mutant animals (Fig. S2A). The deletion is predicted to cause termination of the protein after 35 amino acids from the initial methionine.

Animals from F0, mosaic for the 20-bp deletion in *Wdr45* (indicated with #1 and #2 in Fig. S1), were bred to F1 generation to obtain uniform germline transmission of the mutation. Inbreeding of heterozygous females (*Wdr45*^*−/*+^) with a hemizygous male (*Wdr45*^*−/Y*^) from the F1 generation resulted in hemizygous (*Wdr45*^*−/Y*^, *Wdr45*^+*/Y*^), heterozygous (*Wdr45*^*−/*+^), and homozygous (*Wdr45*^*−/−*^) *Wdr45* KO mice in the F2. By inbreeding F2 animals, we generated two larger *Wdr45* KO cohorts used for in-depth analysis and progression analysis, respectively.

### Quantitative RT-PCR

Total RNA was extracted from brain tissue and primary skin fibroblast cultures established from mice ear clips. AllPrep RNA Kit (Qiagen) was used for the RNA isolation. cDNA was synthesized by the First Strand cDNA synthesis kit (K1612, Thermo) according to the manufacturer’s instructions. Quantitative PCR was performed on a Light Cycler 480 (Roche) using QuantiFast SYBR Green 2X (Qiagen) for the reaction. Primer pairs for *Wdr45* were Forward: GAGGTGTGACCAGCCTACAT and Reverse: CGGTGCAGCATCTCTACC. Primer pairs for *β-actin* were according to (Zhao et al. 2015).

### Study design

Two animal cohorts of mixed background C57BL/6 with FVB were generated and characterized (Fig. S1). The small F2 cohort (21 animals and four genotypes: *Wdr45*^*−/*+^ *n* = 5, *Wdr45*^*−/−*^ *n* = 4, *Wdr45*^*−/Y*^ *n* = 6, *Wdr45*^+*/Y*^ *n* = 6) was only used to investigate the viability and male fertility of *Wdr45* KO mice and evaluate signs of neurological impairment. The medium size “progression cohort” (52 animals: *Wdr45*^*−/*+^ *n* = 10, *Wdr45*^*−/−*^ *n* = 11, *Wdr45*^+*/*+^ *n* = 11, *Wdr45*^*−/Y*^ *n* = 15, *Wdr45*^+*/Y*^ *n* = 12) was employed to evaluate the disease progression in mutant mice over three years and to investigate the mitochondrial function; the large size “phenotyping cohort” consisting of 127 animals older than one year of age (*Wdr45*^*−/*+^ *n* = 25, *Wdr45*^*−/−*^ *n* = 17, *Wdr45*^+*/*+^ *n* = 21, *Wdr45*^*−/Y*^ *n* = 26, *Wdr45*^+*/Y*^ *n* = 38) was provided to the German Mouse Clinic (GMC) for in-depth phenotyping, including statistical analysis of the neurological deterioration (12).

### Mouse housing, nutrition, and handling

Mice were kept in fully air-conditioned experimental animal laboratories at the Helmholtz Zentrum München. The mice’s housing was performed according to FELASA (Federation of European Laboratory Animal Science Associations) guidelines: rooms were kept at a 12/12-h light/dark cycle and in specific pathogen-free conditions. Humidity was set at 50–60%, the temperature at 22 ± 2 °C. Mice were housed up to 4 adults in one cage, in filter top polycarbonate cages type II with 370 cm^2^ surface area, or IVC cages with 501cm^2^ surface areas (Greenline, Tecniplast). Animals were fed with a standard diet for rodents (Altromin, GER) ad libitum.

### Neurobehavioral investigations

All neurobehavioral experiments were performed within the light cycle of the animals. The SHIRPA protocol (Rogers et al. 1997, 2001) was used in its modified scope, according to Masuya and colleagues (Masuya et al. 2005). According to their suggestions, our analysis of the progression cohort included the following experiments:

#### Locomotor activity

Animals were dropped in the middle of an arena (52 × 30 cm, divided into 28 optically distinguishable squares), and quadrants travelled by every single animal in 30 s were counted. This test enabled the assessment of the overall locomotor activity. The Student’s *t* test was used for the statistical analysis of this quantitative variable.

#### Body position

The mouse was placed into a five-litres-viewing jar and observed for a few seconds. Passive behaviour was scored with a 0, active behaviour with a 1, and excessive activity, often including jumping, with a 2. The statistical analysis of this categorical variable was performed using the Student’s *t* test.

#### Tremor

Animals were lifted by the tail, and their forepaws were examined for tremor. Like in humans, a tremor is described as rhythmic muscle contraction that seems to be involuntary. This binary variable was statistically analysed using Fisher’s exact test.

#### Transfer arousal

This is noted when the animal is dropped into the arena for locomotor activity. In the case of an extended freeze (longer than 5 s), the animals score 0 on this test. A shorter freeze resulted in a score of 1 and a rapid movement in a score of 2. This categorical variable was analysed using the Student’s *t* test.

#### Gait

Gait was judged looking at animals walking in the arena. Any abnormality was scored with a 1. Otherwise, a 0 was assigned. This binary variable was analysed using Fisher’s exact test.

#### Tail elevation

Tail elevation was judged by looking at the tail’s position while animals were travelling the arena. Tail elevation was rated 0 when the tail was dragging, 1 when horizontally extended, and 2 when elevated. This categorical variable was analysed using the Student’s *t* test.

#### Clickbox

A clicking sound was applied out of the animals’ sight. Its reaction was measured as either absent or present in order to judge the hearing capability. This binary variable was analysed using Fisher’s exact test.

#### Touch escape

In order to investigate the animals’ reaction to touch, the mouse was frontally approached by an extended finger. In the absence of response, the mouse scored a 0; in the presence of a response, it scored a 1; if the animal fled before touch, it scored a 2.

#### Trunk curl

This observation is assessed while lifting the animal by the tail. Trunk curl describes an instant and straight body curl towards the tail. Trunk curl is often accompanied by the forepaws touching the hindpaws. This binary variable was analysed using Fisher’s exact test since this observation is either absent or present.

#### Limb grasp

This is observed when the animal is lifted by the tail and describes whether the limbs’ grasping is present. As this is also a binary variable, the statistical analysis was performed using Fisher’s exact test.

#### Urination

It was assessed whether urination occurred during the time of SHIRPA performance. Being a binary variable, this was analysed using Fisher’s exact test.

The balance beam was performed in triplicates. All animals were trained to traverse the beams before the first measurement. Four beams of different shapes and diameters were used for the phenotyping cohort (Beams 1 and 3: rectangular, 20 and 12 mm; beams 2 and 4: round, 22, and 15 mm. Only two beams were used for progression analysis (Beam 1: round, 22 mm; beam 2: rectangular, 7 mm). The end of the beam was attached to the mice’s home cage, and the height of the beam was 15 cm, the same height of the cages to encourage traversing of the beam. What was measured was the time it took for the animal to traverse 60 cm of the specific beam and how many foot slips and falls were experienced in each trail.

Grip strength was measured via the grip strength meter (Bioseb, France) in triplicate. We used forepaw measurements if not stated otherwise.

### Social discrimination

The Social Discrimination test was conducted as described previously (Garrett et al. 2015). Briefly, test animals had two 4-min exposures to stimulus animals (ovariectomized 129 Sv females) in a fresh cage to which the test animal had been acclimatized 2 h prior. During the first exposure (sample phase), the test animal was exposed to and allowed to investigate the first stimulus animal (“FAM”). This investigation time-indexed social affinity. During the test phase, after a 2-h retention interval, the test animal was reexposed to the same animal along with a novel unfamiliar animal (“UNFAM”). The difference between the time spent investigating the FAM vs UNFAM stimulus animals was compared using a paired *t* test in both the control and mutant groups. With intact social recognition memory, a difference between the investigation times is evident.

### In-depth phenotyping

In-depth phenotyping was performed within the GMC (mouseclinic.de/) based on the GMC screening pipeline (Fuchs et al. 2012). The customized screening pipeline included different tests, covering all clinically relevant organ systems, with a more in-depth focus on the nervous system. At the age of 46 weeks, a cohort of homozygous, heterozygous, and control female mice (15/15/15) and hemizygous and control male mice (15/15) entered the screening pipeline. Tests in the area of behaviour, neurology, clinical chemistry and haematology, nociception, dysmorphology, allergy, energy metabolism, cardiovascular, eye, and immunology were performed. Mice were sacrificed at 96 weeks of age; subsequently, several organs were submitted to pathologic examination.

Blood samples for clinical chemistry measurements were collected at 14 weeks of age in LI-heparin-coated tubes (Kabe Labortechnik) by puncturing the retrobulbar vein plexus with a glass capillary under isoflurane anaesthesia. An additional aliquot was collected in EDTA-coated tubes for haematological analyses and flow cytometry.

Clinical chemistry analyses were performed using an AU480 clinical chemistry analyzer (Beckman-Coulter) and adapted reagents provided by Beckman-Coulter. Basic haematological values were determined using the Sysmex XT2000iV haematology analyser as described previously (Rathkolb et al. 2013a, b).

Glucose tolerance tests were performed after overnight food deprivation with an intraperitoneal glucose load of 2 g glucose per kilogram body mass after determining the basal blood glucose level. Blood glucose values were determined with a hand-held glucometer from single blood drops collected from the tail vein prior to and 15, 30, 60, and 120 min after glucose application.

If not stated otherwise, data generated by the GMC were analysed using R (Version 3.2.3). Tests for genotype effects were made using t-test, Wilcoxon rank-sum test, linear models, ANOVA and post hoc tests, or Fisher’s exact test depending on the assumed distribution of the parameters and questions addressed by the data. The progression analysis was assessed using a linear mixed-effects model (lme) for age and genotype for sexes independently. A *p* value < 0.05 has been used as a level of significance. A correction for multiple testing has not been performed.

### Animal dissection

Animals were euthanized via CO_2_ or by neck cervical dislocation, according to German directives (TierschutzVersuchstierVerordnung, 1. August 2013). Organ withdrawal was performed in a standardized manner from kidneys, liver, lung, and heart to skeletal muscle and brain as last. Organs were snap-frozen in liquid nitrogen immediately after dissection and stored at -80 °C. For some of the immunohistological analysis, the brain was cut longitudinally into 2 hemispheres, one of which was formalin-fixed and paraffin-embedded.

### Brain pathology and immunohistochemistry

Animals at varying ages from 4 to 18 months (wild-type *n* = 7, *Wdr45*^*−/Y*^ *n* = 13, *Wdr45*^*−/−*^ *n* = 8, *Wdr45*^*−/*+^ *n* = 7) were euthanized and a complete necropsy was performed (André et al. 2018). All organs were fixed in 4% buffered formalin and cut into 4 µm sections for histochemical staining according to standard protocols: haematoxylin and eosin (H&E) staining for the visual examination of histological alterations, Turnbull’s Blue with and without ammonium sulphide, and Perls’ Prussian Blue staining to show iron (both Fe^2+^ and Fe^3+^, only Fe^2+^, and only Fe^3+^, respectively) deposits in spleen and brain, Periodic Acid–Schiff (PAS) staining to check for carbohydrate macromolecules like glycogen or glycoprotein, and Kluver–Barrera staining to mark lipofuscin granules in brain sections.

For immunohistochemistry (IHC), 1-µm-thick coronal sections of the brain were cut, dewaxed, and rehydrated for evaluation of GFAP (Glial fibrillary acidic protein), LC3II (Microtubule-associated protein 1A/1B-light chain phosphatidylethanolamine conjugate), SDHA (Succinate Dehydrogenase Complex Flavoprotein Subunit A), dopamine, and ubiquitin. The following antibodies were used with specified dilutions: polyclonal rabbit antibody against GFAP (Z0334, DakoCytomation, 1:350), monoclonal mouse antibody against LC3II (clone 5F10, 0231–100/LC3-5F10, NanoTools, 1:100), monoclonal mouse antibody against SDHA (14,715, Abcam, 1:1000), monoclonal rat antibody against dopamine (MAB369, Millipore, 1:500), and a polyclonal rabbit antibody against ubiquitin (7780, Abcam, 1:75). IHC was performed using the streptavidin-peroxidase method in an automated immunostainer (DiscoveryXT, Roche). Diaminobenzidine (DAB) was used for the immunohistochemical reaction and haematoxylin as counterstain. Images were made with a slide-scanning system, NanoZoomer 2.0 HT (Hamamatsu Photonics KK, Hamamatsu City, Japan).

### Mitochondrial respiratory chain activities

The protein extraction for the citrate synthase (CS) measurement and OXPHOS enzyme activity was performed as previously described (Feichtinger et al. 2010). Briefly, heart tissues (60–100 mg) and the whole brain were homogenized with a tissue homogenizer (Ultraturrax, IKA, Staufen, Germany) in extraction buffer (20 mM Tris–HCl, pH7.6, 250 mM sucrose, 40 mM KCl, 2 mM EGTA). The samples were further processed with a motor-driven Teflon-glass homogenizer (Potter S, Braun, Melsungen, Germany). The homogenate was finally centrifuged at 600 g for 10 min at 4 °C. The supernatant containing the mitochondrial fraction was used later for the measurement of the enzyme activities or for the western blot analysis. All enzymatic measurements were performed as previously described (Berger et al. 2003; Meierhofer et al. 2004; Feichtinger et al. 2017).

## Supplementary Information

Below is the link to the electronic supplementary material.

Fig. S1 Generation scheme and breeding strategies for Wdr45 KO cohorts. Zygotes were derived from superovulated FVB females mated with C57Bl/6N males (TIFF 749 kb)

Fig. S2 Representation of TALEN sequences targeting Wdr45 and results from the genetic analysis of TALEN-injected founder mice and their offspring. a TALENs pairs are directed against exon 2 of Wdr45 (NM_001290792.1, GRCm38.p6). In green are the 15 bp regions targeted by the designed TALENs. b Lines underneath exon 2 indicate the area deleted in each of the five mutated founder mice (#1-#5). Black arrows point towards the region deleted in individuals #1 and #2. c Sanger sequencing of ear clip-derived DNA from all three genotypically different animal groups. d Quantitative RT-PCR on total RNA extracted from brain tissues of n=3 Wdr45+/+, n=3 Wdr45-/-, n=3 Wdr45+/Y, and n=3 Wdr45-/Y mice and from primary fibroblast cultures established from ear clips of n=3 Wdr45+/Y and n=3 Wdr45-/Y mice. Wdr45 expression is expressed as a fold difference calculated with the 2-DDCt method. Three independent experiments were performed, each with four technical replicates for each sample (TIFF 6213 kb)

Fig. S3 Timeline of neurodegeneration in three homozygous Wdr45-/- females at four months, one year, and 1.5 years of age showing numerous eosinophil spheroids and swollen structures at all ages. Scale bar represents 200 µm (TIFF 1542 kb)

Fig. S4 Altered hearing sensitivity and motor impairment in the phenotyping cohort. a ABR results of the phenotyping cohort (12 months). Wdr45-/Y and Wdr45-/- were virtually deaf at all frequencies tested (Wilcoxon rank-sum test p < 0.001 each, at 30 kHz p<0.05), while Wdr45-/+ still had some hearing capacity. b Grip strength testing (12 months) did not reveal genotype-dependent effects. c Traversing times on beams 1, 2, and 4 were significantly increased in Wdr45-/Y and Wdr45-/- mice (Wilcoxon rank-sum test; p<0.01 each; 12 months). d Increased number of foot slips of Wdr45-/Y as well as Wdr45-/- mice at all beams (Wilcoxon rank-sum test; p<0.01 each). e Traversing time on a beam ladder was also increased for KO mutants (Wilcoxon rank-sum test; p<0.001; 15 months). f, g Forepaw slips on the ladder were not different (f), but hind paw slips were increased for Wdr45-/Y and Wdr45-/- mice (Wilcoxon rank-sum test; p<0.01) (g). The number of mice used for the tests was as follow: Wdr45-/+ n=15, Wdr45-/- n=15, Wdr45+/+ n=15, Wdr45-/Y n=15, Wdr45+/Y n=15. Only in the ladder test Wdr45+/+ n=14 (TIFF 2133 kb)

Fig. S5 Eye imaging of Wdr45 KO mice from the F8-F9 generation: a SD-OCT imaging of the retinal layers showing granular-like signal in female Wdr45-/- and male Wdr45-/Y mice. b SD-OCT evaluation of the total retinal thickness. Test for genotype effect was made using the Wilcoxon rank-sum test (homozygous females/hemizygous males vs wild-type). The number of mice used for the tests was as follows: Wdr45+/+ n=6, Wdr45-/- n=2, Wdr45+/Y n=3 and Wdr45 -/Y n=5 (TIFF 558 kb)

Fig. S6 Clinical chemistry and haematology investigation. TP, albumin, TIBC, β-amylase, triglycerides, iron, transferrin, WBC, RBC, HGB, HCT. Tests for genotype effects were made using the Wilcoxon rank-sum test (homozygous females/hemizygous males vs wild-type). The number of mice used for the tests was as follow: Wdr45-/+ n=15, Wdr45-/- n=14, Wdr45+/+ n=15, Wdr45-/Y n=14, Wdr45+/Y n=15 (TIFF 1354 kb)

Table S1 Ophthalmic biometric parameters from the F4 generation. P-values according to Wilcoxon rank-sum test. The number of mice used for the tests was as follow: Wdr45+/+ n=12, Wdr45-/- n=13, Wdr45-/Y n=13, Wdr45+/Y n=15 (XLSX 12 kb)

Table S2 Clinical chemistry and haematology data. P-values according to Wilcoxon rank-sum test comparing Wdr45-/- female or Wdr45-/Y male mice to respective wild-type controls. The number of mice used for the tests was as follows: Wdr45-/+ n=15, Wdr45-/- n=14, Wdr45+/+ n=15, Wdr45-/Y n=14, Wdr45+/Y n=15 (XLSX 51 kb)

Table S3 Disease phenotypes of Wdr45 KO mice reported in the literature and public repositories (DOCX 29 kb)

## Data Availability

The datasets used and/or analysed during the current study, as well as the animal model generated in the study, are available from the corresponding author on reasonable request.
