# Supplementary figures and images for: A comprehensive phenotypic characterization of a whole-body Wdr45 knock-out mouse
Source: Mamm Genome. 2021 May 27;32(5):332–49. doi: 10.1007/s00335-021-09875-3 (PMC8458197; doi:10.1007/s00335-021-09875-3)

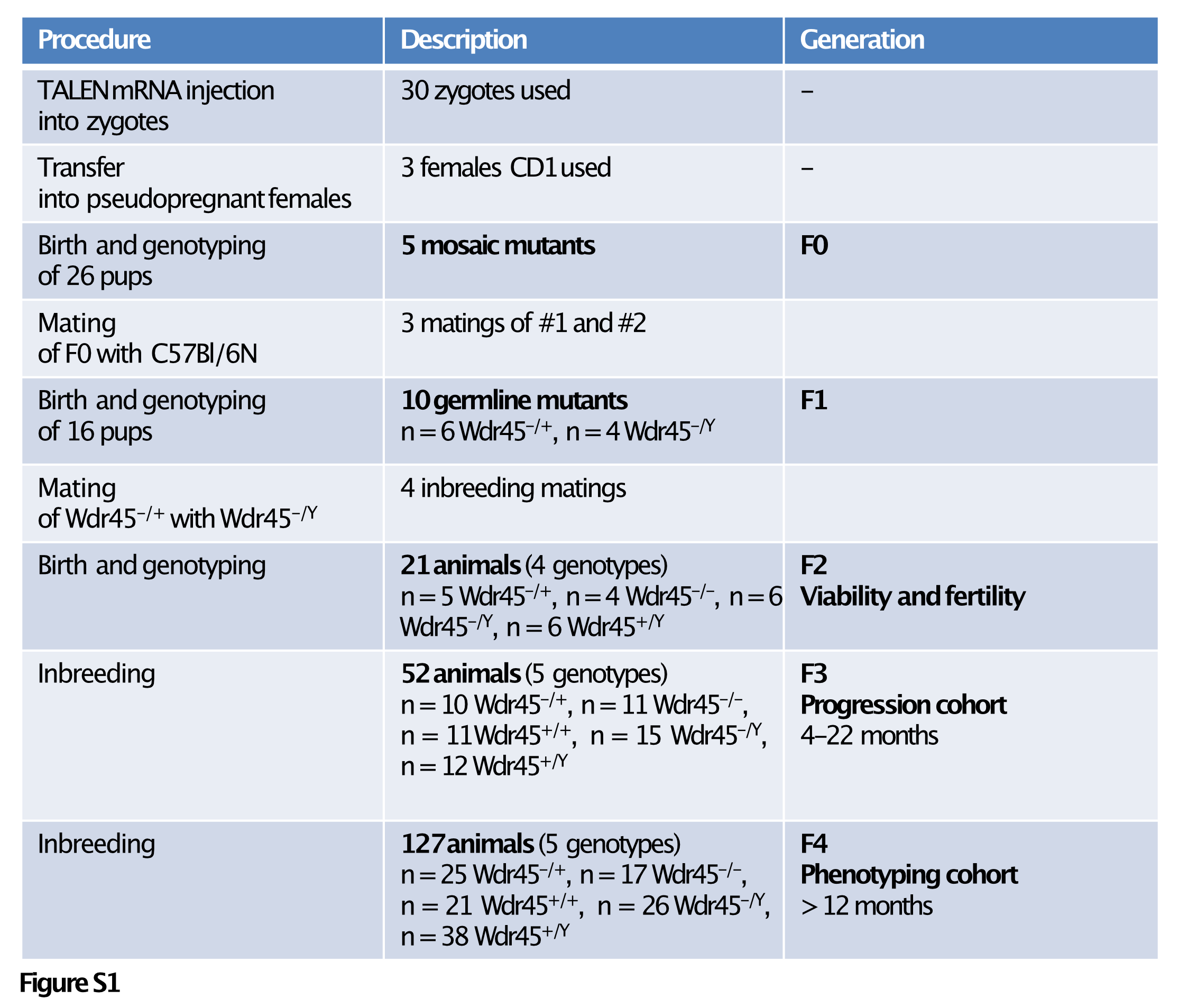

Supplement: Supplementary file 1 — Fig. S1 Generation scheme and breeding strategies for Wdr45 KO cohorts. Zygotes were derived from superovulated FVB females mated with C57Bl/6N males (TIFF 749 kb) [file 335_2021_9875_MOESM1_ESM.tiff]

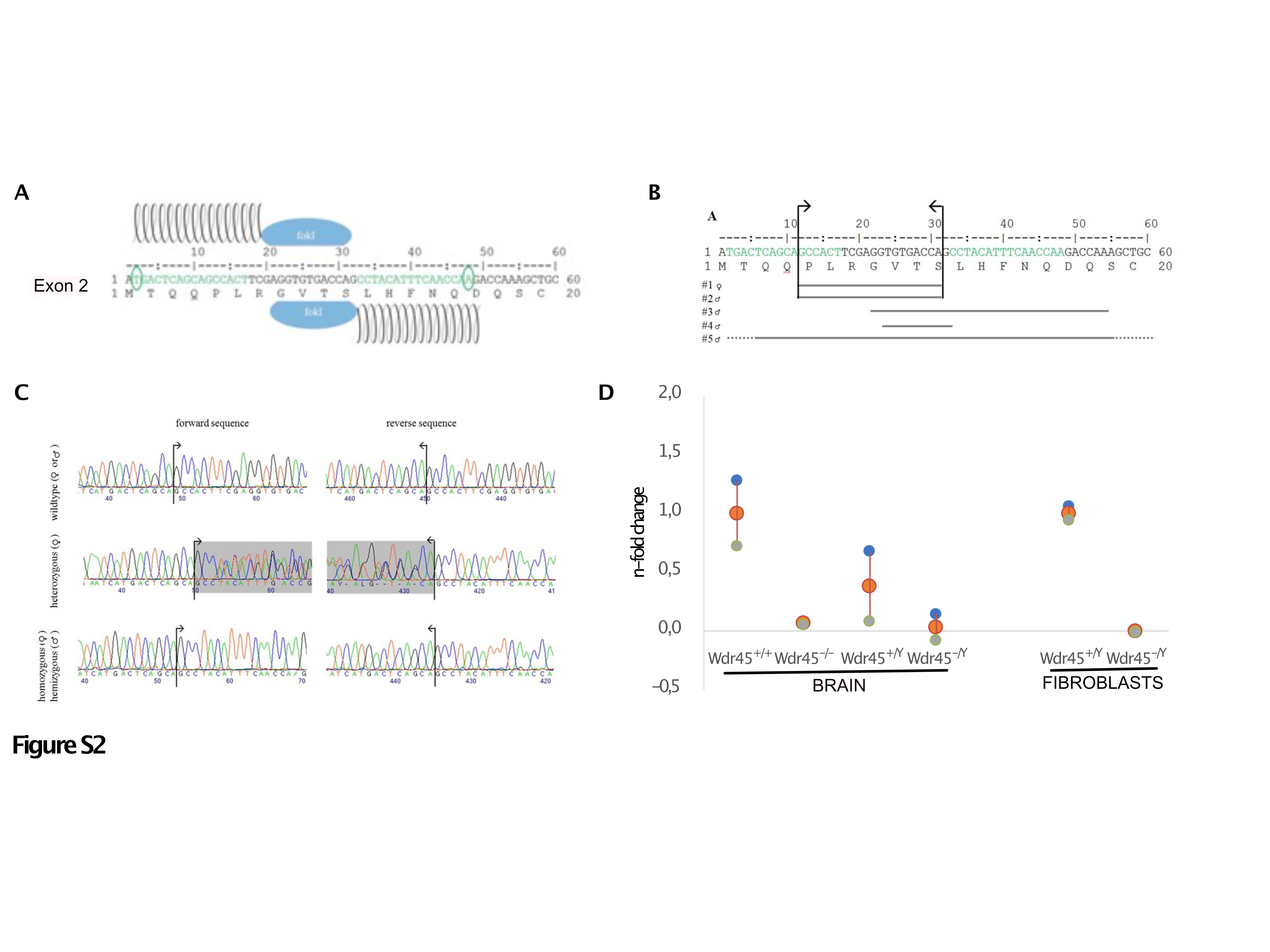

Supplement: Supplementary file 2 — Fig. S2 Representation of TALEN sequences targeting Wdr45 and results from the genetic analysis of TALEN-injected founder mice and their offspring. a TALENs pairs are directed against exon 2 of Wdr45 (NM_001290792.1, GRCm38.p6). In green are the 15 bp regions targeted by the designed TALENs. b Lines underneath exon 2 indicate the area deleted in each of the five mutated founder mice (#1-#5). Black arrows point towards the region deleted in individuals #1 and #2. c Sanger sequencing of ear clip-derived DNA from all three genotypically different animal groups. d Quantitative RT-PCR on total RNA extracted from brain tissues of n=3 Wdr45+/+, n=3 Wdr45-/-, n=3 Wdr45+/Y, and n=3 Wdr45-/Y mice and from primary fibroblast cultures established from ear clips of n=3 Wdr45+/Y and n=3 Wdr45-/Y mice. Wdr45 expression is expressed as a fold difference calculated with the 2-DDCt method. Three independent experiments were performed, each with four technical replicates for each sample (TIFF 6213 kb) [file 335_2021_9875_MOESM2_ESM.tiff]

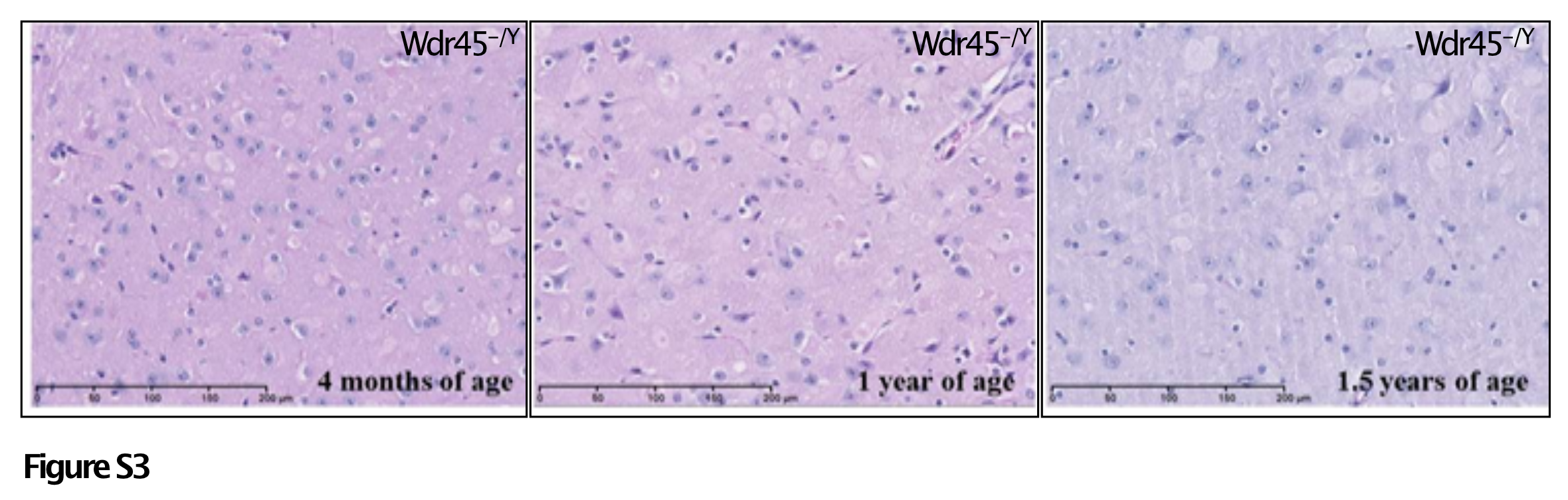

Supplement: Supplementary file 3 — Fig. S3 Timeline of neurodegeneration in three homozygous Wdr45-/- females at four months, one year, and 1.5 years of age showing numerous eosinophil spheroids and swollen structures at all ages. Scale bar represents 200 µm (TIFF 1542 kb) [file 335_2021_9875_MOESM3_ESM.tiff]

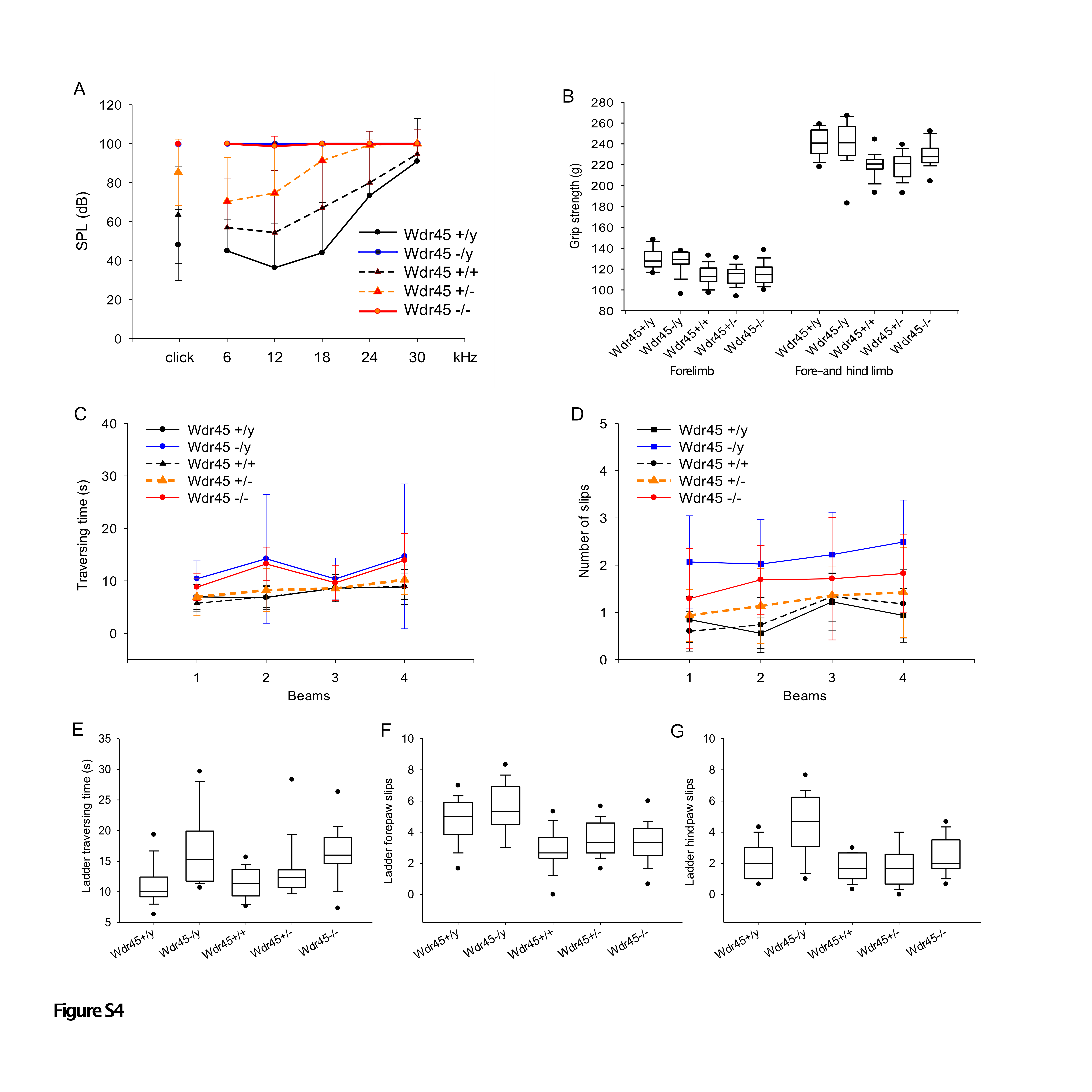

Supplement: Supplementary file 4 — Fig. S4 Altered hearing sensitivity and motor impairment in the phenotyping cohort. a ABR results of the phenotyping cohort (12 months). Wdr45-/Y and Wdr45-/- were virtually deaf at all frequencies tested (Wilcoxon rank-sum test p < 0.001 each, at 30 kHz p<0.05), while Wdr45-/+ still had some hearing capacity. b Grip strength testing (12 months) did not reveal genotype-dependent effects. c Traversing times on beams 1, 2, and 4 were significantly increased in Wdr45-/Y and Wdr45-/- mice (Wilcoxon rank-sum test; p<0.01 each; 12 months). d Increased number of foot slips of Wdr45-/Y as well as Wdr45-/- mice at all beams (Wilcoxon rank-sum test; p<0.01 each). e Traversing time on a beam ladder was also increased for KO mutants (Wilcoxon rank-sum test; p<0.001; 15 months). f, g Forepaw slips on the ladder were not different (f), but hind paw slips were increased for Wdr45-/Y and Wdr45-/- mice (Wilcoxon rank-sum test; p<0.01) (g). The number of mice used for the tests was as follow: Wdr45-/+ n=15, Wdr45-/- n=15, Wdr45+/+ n=15, Wdr45-/Y n=15, Wdr45+/Y n=15. Only in the ladder test Wdr45+/+ n=14 (TIFF 2133 kb) [file 335_2021_9875_MOESM4_ESM.tiff]

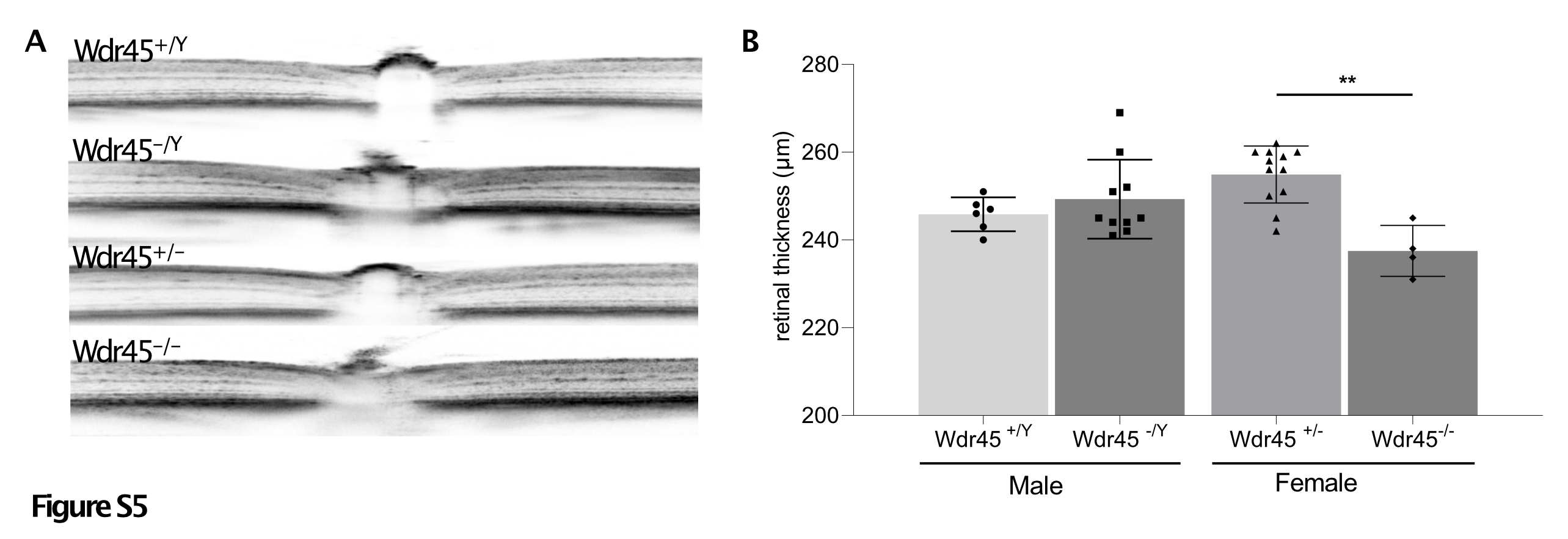

Supplement: Supplementary file 5 — Fig. S5 Eye imaging of Wdr45 KO mice from the F8-F9 generation: a SD-OCT imaging of the retinal layers showing granular-like signal in female Wdr45-/- and male Wdr45-/Y mice. b SD-OCT evaluation of the total retinal thickness. Test for genotype effect was made using the Wilcoxon rank-sum test (homozygous females/hemizygous males vs wild-type). The number of mice used for the tests was as follows: Wdr45+/+ n=6, Wdr45-/- n=2, Wdr45+/Y n=3 and Wdr45 -/Y n=5 (TIFF 558 kb) [file 335_2021_9875_MOESM5_ESM.tiff]

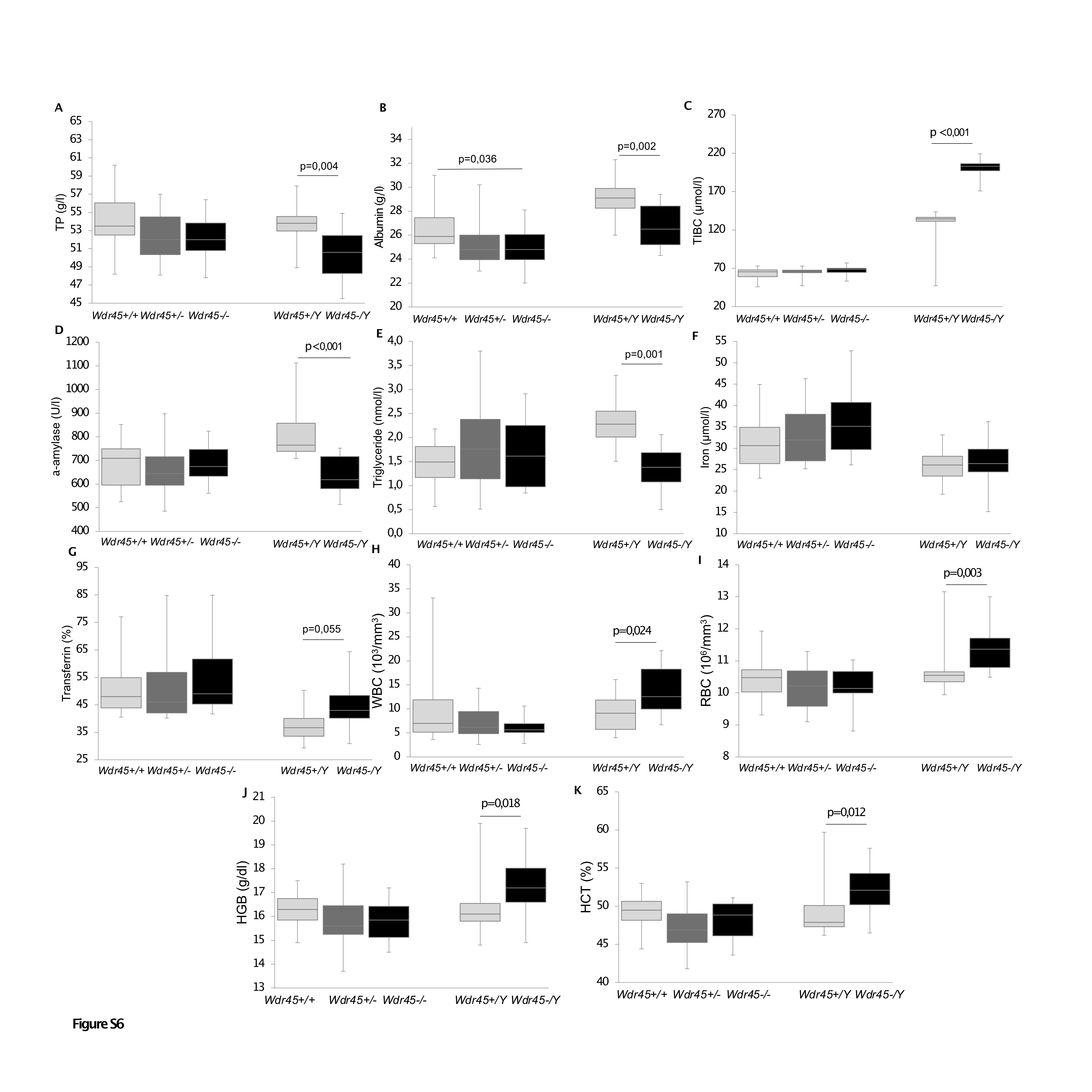

Supplement: Supplementary file 6 — Fig. S6 Clinical chemistry and haematology investigation. TP, albumin, TIBC, β-amylase, triglycerides, iron, transferrin, WBC, RBC, HGB, HCT. Tests for genotype effects were made using the Wilcoxon rank-sum test (homozygous females/hemizygous males vs wild-type). The number of mice used for the tests was as follow: Wdr45-/+ n=15, Wdr45-/- n=14, Wdr45+/+ n=15, Wdr45-/Y n=14, Wdr45+/Y n=15 (TIFF 1354 kb) [file 335_2021_9875_MOESM6_ESM.tiff]
