# Supplementary material for: A comprehensive phenotypic characterization of a whole-body Wdr45 knock-out mouse
Source: Mamm Genome. 2021 May 27;32(5):332–49. doi: 10.1007/s00335-021-09875-3 (PMC8458197; doi:10.1007/s00335-021-09875-3)
Supplement: Supplementary file 9 — Table S3 Disease phenotypes of Wdr45 KO mice reported in the literature and public repositories (DOCX 29 kb) [file 335_2021_9875_MOESM9_ESM.docx]

| **Table S3. Disease phenotypes of Wdr45 KO mice reported in the literature and public repositories.** | | | | | |  |
| --- | --- | --- | --- | --- | --- | --- |
|  |  | **Zhao et al., 2015** | **Wan et al., 2018** | **Biagosch et al.** | **Wdr45^em1(IMPC)J^** | |
| Genetics | Editing technique | Cre-Lox  Conditional CNS-KO (Nes-Wdr45^fl/Y^) | CRISPR-Cas  Constitutive whole-body KO | TALENs  Constitutive whole-body KO | CRISPR-Cas  Constitutive whole-body KO | |
|  | Targeted region | Exons 8-14 | Exon 6 | Exon 2 | Exon 5 | |
|  | Genetic background | 129 R1 x C57BL/6N; back-crossed C57BL/6N | C57BL/6J | mixed FVB x C57BL/6N | C57BL/6N | |
| Neurobehavioral tests | Motor coordination and locomotor activity | **Rotarod**:  Less time spent on the rotarod for KO.  **Morris water maze**: Normal swimming speed.  Age: 11-13 mo.  Sex: Males | **Rotarod**: Tendency to fall for male KO.  Age: 6-8 and 11-13 mo.  Sex: Males and females. Female genotype not specified | **Balance beam:** Increased slips and falls in KO.  Age: 5-22 mo. slips; 18-22 mo. falls.  Sex: Males and females  Increased traversing times in hemi- and homozygous KO.  Age: 12 mo.  Sex: Males and females  **Open field**: Increased locomotor activity and speed in male KO.  Age: 11 mo.  Sex: Males and females | **na** | |
|  | Memory and social discrimination | **Morris water maze**: Impaired retention of spatial memory  **Y-maze**: impaired immediate spatial working memory performances  **Fear conditioning test**: Impaired fear recall.  Age: 11-13 mo.  Sex: Males | **Morris water maze**: Poor learning and memory  **8-arm maze**: Initial increased error rate declining over time  **Fear conditioning test**: Impaired fear recall.  Age: 6 mo.  Sex: Males  **3-chamber test**: No preference for novel conspecific in KO animals.  Age: 6 mo.  Sex: Males and females. Female genotype not specified  **Self-grooming**: Unremarkable  **Marble burying**: Increased in KO.  Age: 2 mo.  Sex: Males and females. Female genotype not specified | **Social discrimination**:  Impaired social recognition memory in male KO.  Age: 12.5 mo.  Sex: Males | **Hole-board Exploration:**  Unremarkable  Age: 2 mo.  Sex: Only male data available | |
|  | Seizure | Spontaneous: na  Induced: na | Spontaneous: na  Induced (pilocarpine): Increased seizure severity in KO  Age: 2 mo.  Sex: Males and females. Female genotype not specified | Spontaneous: Not present  Induced: na | na | |
|  | Body position, passive behaviour, transfer arousal, gait, tail elevation, touch escape, trunk curl, urination | na | na | **modified SHIRPA**: Unremarkable  Age: 5-22 mo.  Sex: Males and females | No difference | |
|  | Grip strength | na | na | Unremarkable | na | |
|  | Tremor | na | na | **modified SHIRPA**: Increased in KO  Age: 20 mo.  Sex: Males and females | No difference | |
|  | Limb grasp | na | na | **modified SHIRPA**: Increased in KO  Age: 18-22 mo.  Sex: Males and females | No difference | |
|  | Hearing | na | na | **ABR:**  Hemi- and homozygous KO no ABR response  Age: 12 mo.  Sex: Males and females  **Modified SHIRPA:** Reduced response to click in KO.  Age: 5-22 mo.  Sex: Males and females | **ABR:**  Hemizygous KO reduced 18 kHz ABR response  Age: 3-4 mo.  Sex: Only male data available  **Modified SHIRPA:** Reduced response to click in KO.  Age: 2 mo.  Sex: Only male data available | |
|  | Vision | na | na | **OCT, eye size:** Retinal degeneration, reduced retinal and cornea thickness, increment of the axial length, deeper anterior chamber  Age: 13 mo., F4 generation; 10-14 mo., F8-9 generations.  Sex: Males and homozygous females | na | |
|  | Electrophysiology | Reduced hippocampal synaptic plasticity | Deficit in the post-synaptic function of the hippocampal synapses. Pre-synaptic are normal | na | na | |
| Additional traits | Bodyweight | na | na | Progression cohort: Higher body weight in all KO.  Age: 22 mo.  Sex: Males and females  Phenotyping cohort:  Higher body weight only in female KO.  Age: 16 mo.  Sex: Males and females | Mild increase of body weight (ns)  Age: 1-4 mo.  Sex: Only male data available | |
|  | Body length, bone mineral content | na | na | **Morphology, X-Ray:** Unremarkable  **DEXA:** na | **Morphology:**  Unremarkable  **DEXA:**  Decreased body length and bone mineral content in homozygous females  Age: 3 mo.  Sex: Only female data available  Reduced bone area in homozygous males  Age: 3 mo.  Sex: Only male data available | |
|  | Body composition | na | na | **Minispec:** Unremarkable | **DEXA:** No difference | |
|  | Energy metabolism |  |  | **Indirect calorimetry:**  Unremarkable | na | |
|  | Cardiovascular parameters | na | na | **Echo and electrocardiography:** Unremarkable | **Electrocardiography:**  Unremarkable | |
|  | Immunological reactions/allergies | na | na | Immunoglobulins, IgE levels, TEWL, Body surface temperature: Unremarkable | na | |
|  | Clinical chemistry | na | na | Increased plasma creatinine, glucose, ASAT, LDH, ALP, and decreased plasma lactate in hemi and homozygous KO.  A similar trend in heterozygous female KO, although the difference was statistically ns.  Decreased TP, albumin, TIBC in all KO and decreased alpha-amylase activity and triglycerides levels in male KO. Slightly increased plasma iron concentration and increased transferrin saturation in KO.  Glucose tolerance test: mild delay of glucose clearance in homozygous KO females; same trend in heterozygous females. Ns differences in males.  Age: 14 mo.  Sex: Males and females | na | |
|  | Haematology | na | na | Elevated RDW in all KO.  Elevated WBC, RBC, HGB HCT in male KO  Age: 14 mo.  Sex: Males and females | na | |
| Histology and Immunofluorescence staining | Iron | Method not specified: Not present in the brain  Age: na  Sex: na | Perls’ Prussian Blue staining: Present in KO brains.  Age: 16 mo.  Sex: Males and females. Female genotype not specified | Turnbull’s Blue with/without APS; Perls’ Prussian Blue: Not present in brains and erythropoiesis organs  Age: 18 mo.  Sex: Male and females | na | |
|  | H&E | Axon swellings in cortex, thalamus, hypothalamus, deep cerebellar nuclei  Vacuolated structures in the thalamus, inferior colliculus, medulla  Progressive eosinophilic spheroids between 6 weeks and 13 mo.  Age: 13 mo.  Sex: Male and females, both genotypes | na | Spheroids and swollen structures (degenerated neurons) in basal ganglia, thalamus, cerebral cortex, medulla oblongata, ascending and descending fibres of the spinal cord and deep cerebellar nuclei  Progressive eosinophilic spheroids between 4- 18 mo.  Age: 4-18 mo.  Sex: Males | na | |
|  | Ultrastructural analysis | Swollen mitochondria in axons of the deep cerebellar nuclei  Demyelinated axons  Age: 13 mo.  Sex: Male | Enlarged ER tubules in KO mice  Mitochondria accumulation in swollen axon  Age: 7 mo.  Sex: Males | na | na | |
|  | Immunohistochemistry | **p62 and ubiquitin**: Accumulation of aggregates in the thalamus, deep cerebellar nuclei, medulla  **GFAP**: Astrogliosis in hippocampus, cortex  **CALB**: Purkinje cell axonal swellings  Age: 13 mo.  Sex: Male and females, both genotypes | **TH:** Reduced TH^+^ neurons in SNR  **RBFOX3:** Reduced RBFOX3^+^ neurons in the pre-frontal cortex  **TUNEL assay**: elevated TUNEL labelling in the pre-frontal cortex  Age: 16 mo.  Sex: Not specified  **HSPA5**: Increased in SNR  Age: 8 mo.  Sex: Not specified | **Ubiquitin**: Accumulation of aggregates in the medulla oblongata  **GFAP**: Accumulation of aggregates in the medulla oblongata  **CALB**: impaired Purkinje cell layer of the cerebellar cortex  **Dopamine**: reduced number of neuroaxonal fibres in the substantia nigra  **Kluver-Barrera staining**: No lipofuscin accumulation  **PAS staining:** No polysaccharides and glycolipids accumulation  Age: 18 mo.  Sex: Males | na | |
| na: Not available; mo: Months; ns: Not significant | | | | | | |
